# Supplementary material for: Revisiting Fur Regulon Leads to a Comprehensive Understanding of Iron and Fur Regulation
Source: Int J Mol Sci. 2023 May 22;24(10):9078. doi: 10.3390/ijms24109078 (PMC10219339; doi:10.3390/ijms24109078)
Supplement: Supplementary file 1 [file ijms-24-09078-s001.zip › ijms-2323480-supplementary.pdf]

Supplementary Materials

Revisiting Fur regulon leads to a comprehensive understanding of iron and Fur regulation

Chaofan Hou [†], Lin Liu [†], Xian Ju , Yunzhu Xiao , Bingyu Li , Conghui You*

Shenzhen Key Laboratory of Microbial Genetic Engineering, College of Life Sciences and Oceanology, Shenzhen University, Shenzhen 518055, China

*Correspondence: cyou@szu.edu.cn

[†] These authors contributed equally to this work.

Supplementary Materials

Supplementary Figures

Figure S1.Binding peak of Fur identified upstream of *flhDC*.

Figure S2. Expression of motility genes by RT-qPCR.

Figure S3. Characterization of Fur regulation on motility in *E. coli* K12 strain MG1655.

Supplementary Tables

Table S1. DEGs identified comparing the transcriptomes of NCM3722 (Fur+) grown in the medium with iron versus that without iron.

Table S2. DEGs identified comparing the transcriptomes of NCM3722 (Fur+) versus that of CY405 (Fur-) grown in the medium with iron supplied.

Table S3. DEGs identified comparing the transcriptomes of NCM3722 (Fur+) versus that of CY405 (Fur-) grown in the medium without iron supplied.

Table S4. Binding peaks of Fur identified in this work.

Table S5. Transcriptional units identified to be controlled by Fur directly in this study.

Table S6. Predicated binding sites of Fur on genes repressed by Fur.

Table S7. Predicated binding sites of Fur on genes activated by Fur.

Table S8. Fold changes of DEGs directly controlled by Fur identified in the transcriptome comparison of CY405 versus NCM3722 grown in the conditions with or without iron.

Table S9. Fold changes of DEGs directly controlled by Fur identified in the transcriptome comparison of CY405 versus NCM3722 grown in the condition with iron.

Table S10. Strains used in this study.

Table S11. Primers used in EMSA assay in this study.

Table S12. Primers of RT-qPCR used in this study.

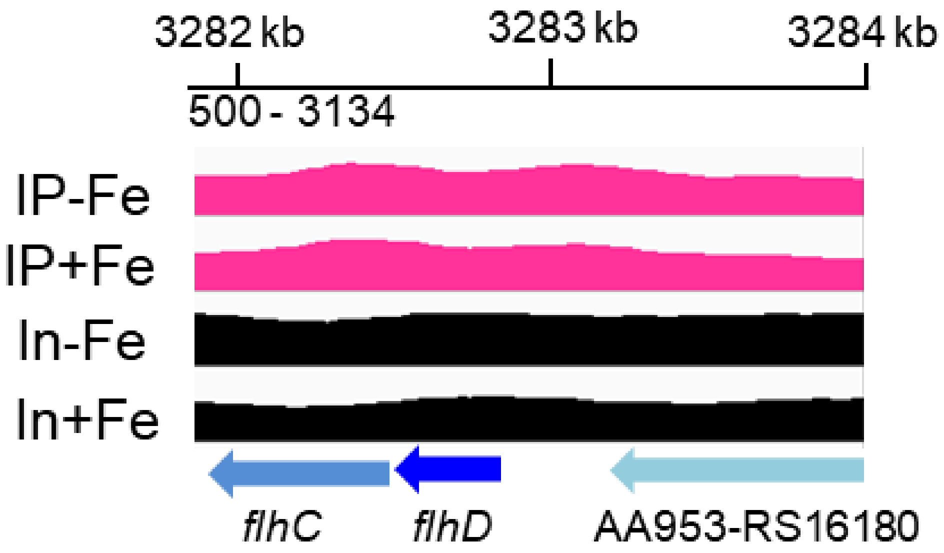

Figure S1. Binding peak of Fur identified upstream of *flhDC*.
This is the IGV image of the Fur binding profiles in the chromosomal region near *flhDC*. IP-Fe/In-Fe: ChIP-seq (IP-Fe) or input control (In-Fe) result in the growth condition without iron; IP+Fe/In+Fe: ChIP-seq (IP+Fe) or input control (In+Fe) result in the growth condition with iron. “500 – 3134” indicates the range of the scale.

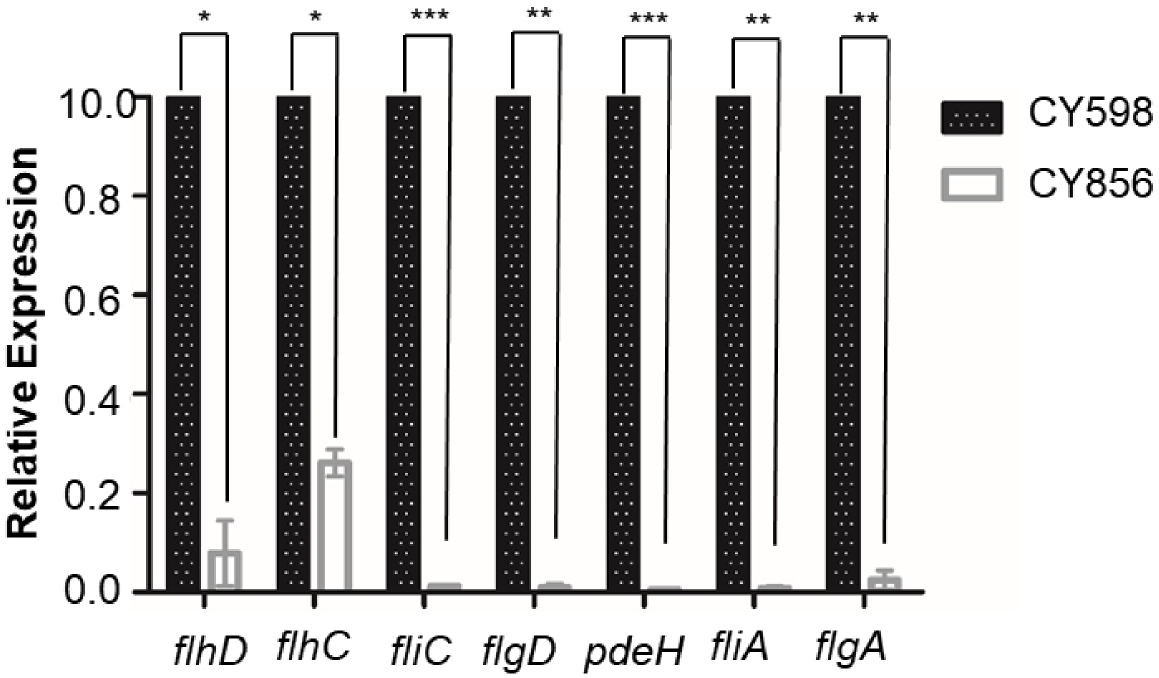

Figure S2. **Expression of motility genes by RT-qPCR.**

The expression of *flhDC* together with several motility genes in the two strains was detected by RT-qPCR. The mRNA level of each gene in CY598 was normalized to 1 and that in CY856 was determined relative to this value. The relative expression was shown as the average ± S.D. of three independent experiments. *, $p \leq 0.05$; **, $p \leq 0.01$; ***, $p \leq 0.001$.

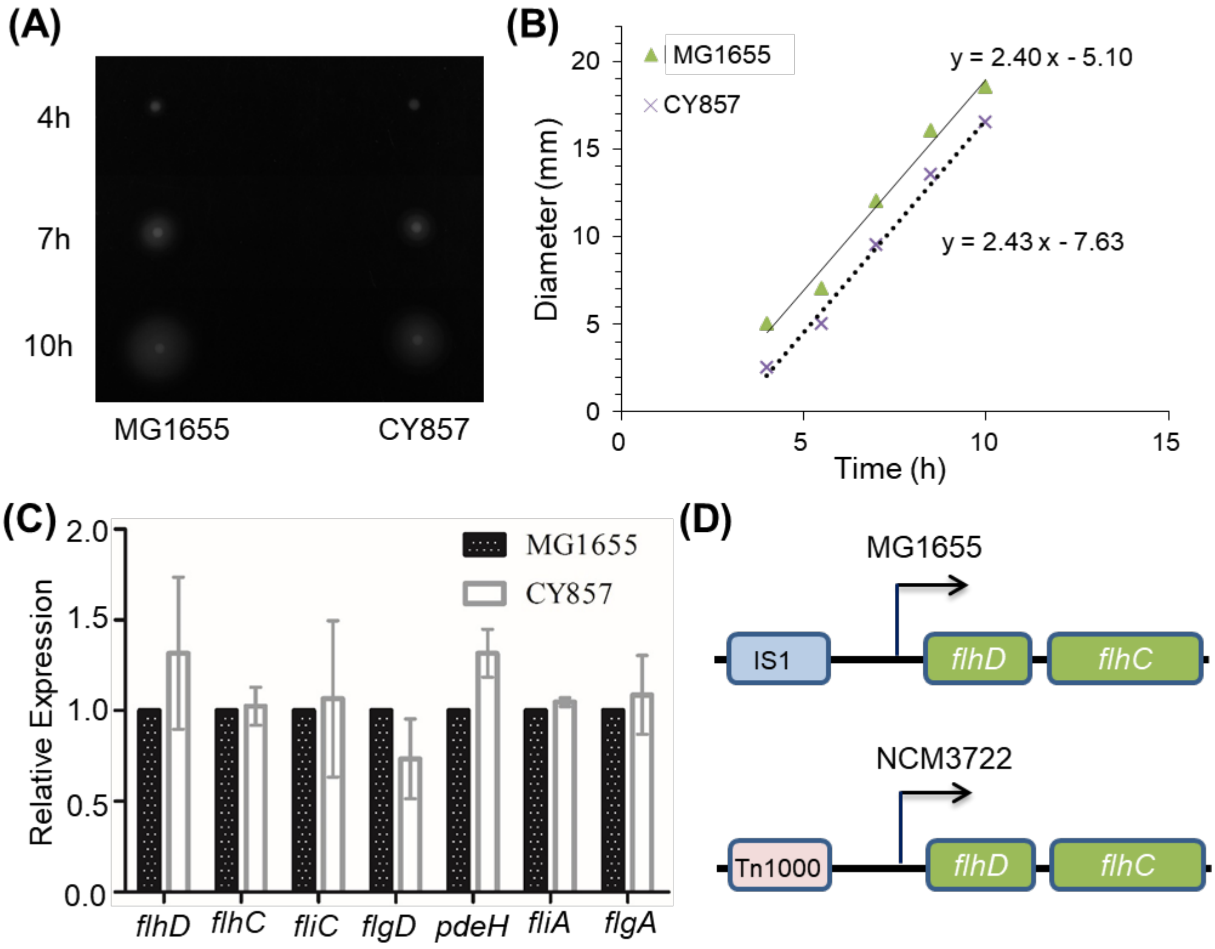

Figure S3. **Characterization of Fur regulation on motility in *E. coli* K12 strain MG1655.** (A) Swimming zones of the two strains on soft-agar plates after incubation at 37 °C for different time. CY857 is the Fur knockout strain derived from MG1655. Two independent cultures were tested and one representative image is shown. (B) Swimming speed of the two strains on soft-agar plates. The diameter of the swimming zone in (A) was plotted against time. The slope of the linear fitting indicates swimming speed (mm/h). (C) The expression of *flhDC* together with several motility genes in the two strains was detected by RT-qPCR. The mRNA level of each gene in MG1655 was normalized to 1 and that in CY857 was determined relative to this value. The relative expression was shown as the average ± S.D. of three independent experiments. (D) Illustration of the upstream of the *flhDC* operon in MG1655 and NCM3722 strains. Insertion sequences of IS1 and Tn1000 are respectively located upstream of the *flhDC* operon in the two strains which may change the regulatory region in different ways.

Table S1. DEGs identified comparing the transcriptomes of NCM3722 (Fur+) grown in the medium with iron versus that without iron.

Gene ID	Gene Symbol	Log_2 FC (Fe+ vs Fe-)	*q*-value	Diff
AA953_RS00160	*rplF*	-0.61	0.0052	Down
AA953_RS00170	*rpsN*	-0.47	0.0042	Down
AA953_RS00225	*rplW*	-0.53	0.0002	Down
AA953_RS00690	*feoB*	-0.65	9.67E-05	Down
AA953_RS02920	*metE*	-0.95	4.24E-05	Down
AA953_RS05325	*fecA*	-1.27	0.0022	Down
AA953_RS05775	*bglJ*	-0.93	0.0009	Down
AA953_RS05780	*fhuF*	-1.23	0.0003	Down
AA953_RS05785	*yjjZ*	-2.67	0.0007	Down
AA953_RS06740	*fhuA*	-0.99	1.84E-07	Down
AA953_RS06745	*fhuC*	-1.17	1.42E-08	Down
AA953_RS06750	*fhuD*	-1.05	0.0009	Down
AA953_RS06755	*fhuB*	-0.94	0.0004	Down
AA953_RS08940	*entD*	-3.36	0.0004	Down
AA953_RS08945	*fepA*	-4.47	1.84E-07	Down
AA953_RS08950	*fes*	-3.41	4.47E-05	Down
AA953_RS08960	*entF*	-3.57	7.21E-07	Down
AA953_RS08970	*fepC*	-1.64	1.05E-06	Down
AA953_RS08975	*fepG*	-1.59	7.38E-06	Down
AA953_RS08980	*fepD*	-1.22	5.38E-05	Down
AA953_RS08985	*entS*	-1.61	6.63E-09	Down
AA953_RS08990	*fepB*	-1.61	8.72E-09	Down
AA953_RS08995	*entC*	-3.67	3.45E-07	Down
AA953_RS09000	*entE*	-3.88	2.10E-09	Down
AA953_RS09005	*entB*	-3.84	1.49E-09	Down
AA953_RS09010	*entA*	-3.77	1.34E-09	Down
AA953_RS09015	*entH*	-3.33	0.0001	Down
AA953_RS09865	*modA*	-1.49	1.99E-15	Down
AA953_RS09870	*modB*	-1.66	1.42E-08	Down
AA953_RS09875	*modC*	-1.28	7.44E-07	Down
AA953_RS10420	*ybiX*	-3.54	4.13E-06	Down
AA953_RS10425	*fiu*	-4.25	2.43E-06	Down
AA953_RS11555	*efeO*	-1.04	1.24E-06	Down
AA953_RS11990	*fhuE*	-2.94	0.0001	Down
AA953_RS13865	*yncE*	-1.83	7.35E-09	Down
AA953_RS14075	*pqqL*	-1.19	3.87E-07	Down
AA953_RS14080	*yddB*	-1.17	4.03E-06	Down
AA953_RS15040	*sufE*	-1.35	8.63E-06	Down
AA953_RS15045	*sufS*	-1.58	6.76E-11	Down
AA953_RS15050	*sufD*	-1.79	7.65E-11	Down
AA953_RS15055	*sufC*	-1.59	6.96E-08	Down

AA953_RS15060	*sufB*	-1.77	1.96E-09	Down
AA953_RS15065	*sufA*	-1.48	6.63E-09	Down
AA953_RS15180	*ydiE*	-1.26	0.0015	Down
AA953_RS15775	*manY*	-0.62	1.24E-05	Down
AA953_RS15780	*manZ*	-0.46	0.0015	Down
AA953_RS16620	*shiA*	-0.96	0.003	Down
AA953_RS16830	*hisD*	-0.63	0.0033	Down
AA953_RS17155	*thiD*	-0.64	0.0015	Down
AA953_RS17435	*cirA*	-4.49	3.45E-07	Down
AA953_RS20065	*gabP*	-0.66	0.0009	Down
AA953_RS20125	*nrdE*	-1.97	0.002	Down
AA953_RS21405	*pgk*	-0.53	0.0005	Down
AA953_RS21840	*exbD*	-0.91	0.0033	Down
AA953_RS21845	*exbB*	-1.05	9.45E-05	Down
AA953_RS22185	*yqjH*	-0.93	5.73E-05	Down
AA953_RS00315	*bfr*	0.98	0.0016	up
AA953_RS14910	*grxD*	0.64	0.0099	up

Table S2. DEGs identified comparing the transcriptomes of NCM3722 (Fur+) versus that of CY405 (Fur-) grown in the medium with iron supplied.

Gene ID	Gene Symbol	Log_2 FC (Fur+ vs Fur-)	*q-value*	Diff
AA953_RS00085	*mscL*	-0.41	0.0073	Down
AA953_RS00320	*bfd*	-1.49	4.69E-08	Down
AA953_RS00715	*nfuA*	-0.52	8.93E-06	Down
AA953_RS01160	*dinQ*	-1.11	3.39E-09	Down
AA953_RS01215	*hdeB*	-0.67	0.0053	Down
AA953_RS01745	*lldP*	-0.44	0.003	Down
AA953_RS01755	*lldD*	-0.51	0.0001	Down
AA953_RS01955	*dinD*	-1.15	5.33E-08	Down
AA953_RS02115	*AA953_RS02115*	-2.39	1.13E-17	Down
AA953_RS02120	*tisB*	-2.59	6.51E-15	Down
AA953_RS02335	*chrR*	-0.64	4.16E-07	Down
AA953_RS02640	*ilvC*	-0.82	1.20E-09	Down
AA953_RS02840	*uvrD*	-0.44	0.0014	Down
AA953_RS02935	*rmuC*	-0.64	7.37E-06	Down
AA953_RS03385	*tpiA*	-0.67	2.21E-07	Down
AA953_RS03410	*fpr*	-0.63	0.0001	Down
AA953_RS03750	*thiH*	-0.86	5.94E-08	Down
AA953_RS03755	*thiG*	-0.95	9.80E-08	Down
AA953_RS03765	*thiF*	-0.94	1.72E-06	Down
AA953_RS03770	*thiE*	-0.94	9.20E-12	Down
AA953_RS03775	*thiC*	-0.90	3.26E-15	Down
AA953_RS03930	*yjbD*	-0.41	0.0029	Down
AA953_RS03935	*lysC*	-0.67	8.59E-06	Down
AA953_RS03940	*pgi*	-0.42	0.0022	Down
AA953_RS03945	*yjbE*	-1.36	6.29E-08	Down
AA953_RS04050	*lexA*	-1.18	6.75E-23	Down
AA953_RS04055	*dinF*	-1.00	2.42E-06	Down
AA953_RS04090	*qorA*	-0.56	0.0004	Down
AA953_RS04130	*uvrA*	-0.78	2.03E-06	Down
AA953_RS04150	*soxS*	-1.51	1.23E-22	Down
AA953_RS04925	*ytfE*	-1.79	9.39E-08	Down
AA953_RS05010	*ppa*	-0.48	0.0007	Down
AA953_RS05305	*fecE*	-1.31	5.44E-06	Down
AA953_RS05310	*fecD*	-1.17	0.0025	Down
AA953_RS05315	*fecC*	-1.35	3.21E-05	Down
AA953_RS05320	*fecB*	-1.28	1.92E-06	Down
AA953_RS05325	*fecA*	-2.34	3.87E-53	Down
AA953_RS05330	*fecR*	-3.08	8.82E-113	Down
AA953_RS05335	*fecI*	-3.32	2.26E-88	Down
AA953_RS05570	*AA953_RS05570*	-2.08	4.43E-07	Down

AA953_RS05575	*iraD*	-1.41	0.0019	Down
AA953_RS05645	*yjiR*	-0.68	7.62E-05	Down
AA953_RS05675	*symE*	-1.47	0.001	Down
AA953_RS05775	*bglJ*	-1.73	5.35E-21	Down
AA953_RS05780	*fhuF*	-1.84	5.86E-35	Down
AA953_RS05785	*yjjZ*	-7.22	0	Down
AA953_RS06225	*folA*	-0.47	0.0009	Down
AA953_RS06280	*polB*	-0.86	4.77E-09	Down
AA953_RS06320	*tbpA*	-0.46	0.0008	Down
AA953_RS06340	*leuD*	-0.64	3.76E-07	Down
AA953_RS06345	*leuC*	-0.72	0.0008	Down
AA953_RS06350	*leuB*	-0.57	0.0006	Down
AA953_RS06355	*leuA*	-0.80	9.85E-07	Down
AA953_RS06505	*guaC*	-0.49	0.0004	Down
AA953_RS06655	*panB*	-0.37	0.0027	Down
AA953_RS06740	*fhuA*	-1.06	5.24E-18	Down
AA953_RS06745	*fhuC*	-1.19	1.43E-12	Down
AA953_RS06750	*fhuD*	-1.14	5.94E-08	Down
AA953_RS06755	*fhuB*	-0.73	0.0013	Down
AA953_RS07025	*dkgB*	-1.43	1.50E-15	Down
AA953_RS07100	*ivy*	-0.94	3.16E-14	Down
AA953_RS07155	*dinB*	-1.53	9.09E-12	Down
AA953_RS07825	*hemB*	-0.32	0.0092	Down
AA953_RS07930	*aroL*	-0.42	0.0009	Down
AA953_RS07960	*rdgC*	-0.55	0.0002	Down
AA953_RS08125	*yajQ*	-0.53	0.0006	Down
AA953_RS08135	*cyoE*	-0.60	0.0099	Down
AA953_RS08145	*cyoC*	-0.78	0.0003	Down
AA953_RS08150	*cyoB*	-0.62	0.0004	Down
AA953_RS08155	*cyoA*	-0.53	4.47E-06	Down
AA953_RS08340	*ybaN*	-1.16	1.01E-06	Down
AA953_RS08810	*nohD2*	-3.21	5.58E-37	Down
AA953_RS08835	*ompT*	-0.60	6.46E-07	Down
AA953_RS08905	*nfsB*	-0.47	0.0008	Down
AA953_RS08940	*entD*	-4.64	1.40E-105	Down
AA953_RS08945	*fepA*	-5.74	3.81E-34	Down
AA953_RS08950	*fes*	-4.56	4.44E-153	Down
AA953_RS08955	*ybdZ*	-5.28	1.81E-09	Down
AA953_RS08960	*entF*	-4.71	5.71E-128	Down
AA953_RS08965	*fepE*	-2.28	1.40E-11	Down
AA953_RS08970	*fepC*	-2.10	6.50E-33	Down
AA953_RS08975	*fepG*	-1.91	2.76E-25	Down
AA953_RS08980	*fepD*	-1.65	7.73E-32	Down
AA953_RS08985	*entS*	-1.97	3.49E-43	Down

AA953_RS08990	*fepB*	-2.41	1.61E-120	Down
AA953_RS08995	*entC*	-4.91	7.77E-35	Down
AA953_RS09000	*entE*	-4.82	2.03E-136	Down
AA953_RS09005	*entB*	-4.66	1.86E-150	Down
AA953_RS09010	*entA*	-4.52	1.20E-169	Down
AA953_RS09015	*entH*	-4.22	1.70E-79	Down
AA953_RS09070	*uspG*	-0.77	5.33E-09	Down
AA953_RS09430	*uof*	-1.92	2.33E-08	Down
AA953_RS09440	*fldA*	-0.51	8.17E-05	Down
AA953_RS09630	*gltA*	-1.02	2.80E-15	Down
AA953_RS09635	*AA953_RS09635*	-1.04	5.91E-13	Down
AA953_RS09795	*nadA*	-0.49	0.0004	Down
AA953_RS09800	*pnuC*	-0.38	0.0083	Down
AA953_RS09825	*gpmA*	-2.20	2.72E-82	Down
AA953_RS09850	*modF*	-0.48	6.77E-05	Down
AA953_RS09855	*modE*	-0.60	0.0023	Down
AA953_RS09880	*ybhA*	-0.74	6.75E-06	Down
AA953_RS09890	*ybhD*	-1.43	3.53E-10	Down
AA953_RS09895	*ybhH*	-2.17	0.0008	Down
AA953_RS09915	*AA953_RS09915*	-1.85	3.84E-27	Down
AA953_RS09920	*exc*	-3.03	0.0006	Down
AA953_RS09925	*AA953_RS09925*	-2.60	2.87E-08	Down
AA953_RS09930	*AA953_RS09930*	-0.82	1.72E-09	Down
AA953_RS09935	*AA953_RS09935*	-2.47	6.89E-33	Down
AA953_RS09940	*TraR*	-3.25	9.39E-08	Down
AA953_RS09965	*yqaJ*	-2.22	2.04E-08	Down
AA953_RS09970	*AA953_RS09970*	-2.74	7.17E-36	Down
AA953_RS09975	*gam*	-2.34	9.49E-17	Down
AA953_RS09985	*AA953_RS09985*	-3.73	0.0003	Down
AA953_RS09990	*AA953_RS09990*	-2.89	3.45E-50	Down
AA953_RS09995	*AA953_RS09995*	-2.63	3.04E-41	Down
AA953_RS10000	*AA953_RS10000*	-1.60	2.80E-15	Down
AA953_RS10005	*AA953_RS10005*	-2.50	4.82E-43	Down
AA953_RS10015	*rexB*	-0.95	1.04E-09	Down
AA953_RS10020	*rexA*	-0.53	0.0011	Down
AA953_RS10025	*AA953_RS10025*	-0.48	0.0002	Down
AA953_RS10030	*AA953_RS10030*	-3.21	1.10E-68	Down
AA953_RS10035	*AA953_RS10035*	-2.25	1.46E-14	Down
AA953_RS10040	*AA953_RS10040*	-2.88	6.19E-44	Down
AA953_RS10045	*AA953_RS10045*	-3.38	6.05E-34	Down
AA953_RS10050	*renD*	-2.09	7.99E-05	Down
AA953_RS10055	*AA953_RS10055*	-3.41	1.65E-11	Down
AA953_RS10060	*AA953_RS10060*	-3.00	5.41E-23	Down
AA953_RS10075	*ninG*	-4.06	2.62E-21	Down

AA953_RS10085	*ninI*	-3.50	2.16E-40	Down
AA953_RS10090	*AA953_RS10090*	-2.97	5.44E-40	Down
AA953_RS10095	*AA953_RS10095*	-3.59	1.43E-63	Down
AA953_RS10100	*AA953_RS10100*	-3.47	1.54E-64	Down
AA953_RS10105	*rzpD2*	-2.07	3.66E-42	Down
AA953_RS10110	*borD2*	-0.49	0.0036	Down
AA953_RS10120	*ybcV2*	-2.40	1.29E-05	Down
AA953_RS10125	*ybcW*	-2.73	4.94E-11	Down
AA953_RS10130	*nohD*	-3.57	2.24E-08	Down
AA953_RS10135	*ybcX*	-3.50	1.91E-54	Down
AA953_RS10145	*AA953_RS10145*	-3.57	1.77E-80	Down
AA953_RS10150	*AA953_RS10150*	-3.66	1.03E-92	Down
AA953_RS10155	*AA953_RS10155*	-3.38	2.86E-84	Down
AA953_RS10160	*AA953_RS10160*	-3.64	2.19E-104	Down
AA953_RS10165	*AA953_RS10165*	-3.96	8.45E-47	Down
AA953_RS10170	*AA953_RS10170*	-3.01	3.27E-15	Down
AA953_RS10175	*AA953_RS10175*	-3.79	1.08E-46	Down
AA953_RS10180	*AA953_RS10180*	-3.74	1.87E-33	Down
AA953_RS10185	*AA953_RS10185*	-3.55	1.66E-70	Down
AA953_RS10190	*AA953_RS10190*	-3.47	1.86E-54	Down
AA953_RS10195	*AA953_RS10195*	-3.47	1.85E-34	Down
AA953_RS10200	*AA953_RS10200*	-3.45	8.38E-77	Down
AA953_RS10205	*AA953_RS10205*	-3.68	3.88E-07	Down
AA953_RS10210	*AA953_RS10210*	-3.28	1.14E-44	Down
AA953_RS10215	*AA953_RS10215*	-3.38	1.81E-23	Down
AA953_RS10220	*AA953_RS10220*	-3.54	6.34E-42	Down
AA953_RS10225	*AA953_RS10225*	-3.48	2.75E-77	Down
AA953_RS10230	*AA953_RS10230*	-2.80	1.36E-43	Down
AA953_RS10235	*AA953_RS10235*	-3.65	5.86E-75	Down
AA953_RS10240	*tfaQ2*	-3.31	6.64E-32	Down
AA953_RS10250	*AA953_RS10250*	-1.29	9.92E-09	Down
AA953_RS10260	*ybhB*	-0.79	1.06E-09	Down
AA953_RS10265	*bioA*	-1.69	3.45E-53	Down
AA953_RS10270	*bioB*	-0.81	3.97E-08	Down
AA953_RS10275	*bioF*	-1.13	1.20E-15	Down
AA953_RS10280	*bioC*	-0.73	1.12E-05	Down
AA953_RS10285	*bioD*	-0.79	2.51E-09	Down
AA953_RS10290	*uvrB*	-0.84	7.42E-13	Down
AA953_RS10300	*AA953_RS10300*	-2.15	2.56E-11	Down
AA953_RS10305	*moaA*	-1.95	1.72E-78	Down
AA953_RS10310	*moaB*	-1.97	2.38E-52	Down
AA953_RS10315	*moaC*	-1.71	2.73E-31	Down
AA953_RS10320	*moaD*	-2.03	0.0002	Down
AA953_RS10325	*moaE*	-1.39	3.41E-10	Down

AA953_RS10395	*dinG*	-0.71	4.56E-06	Down
AA953_RS10410	*ybiJ*	-0.91	0.0004	Down
AA953_RS10415	*ybiI*	-1.25	9.78E-07	Down
AA953_RS10420	*ybiX*	-4.68	7.04E-123	Down
AA953_RS10425	*fiu*	-5.14	1.58E-167	Down
AA953_RS10430	*mcbA*	-0.70	0.0023	Down
AA953_RS10475	*ompX*	-0.92	2.52E-15	Down
AA953_RS10515	*ybiV*	-0.54	0.0008	Down
AA953_RS10660	*nfsA*	-0.66	0.0028	Down
AA953_RS10665	*rimK*	-0.51	0.0092	Down
AA953_RS10765	*poxB*	-0.64	1.37E-06	Down
AA953_RS10910	*ycaC*	-0.51	0.0008	Down
AA953_RS11155	*pyrD*	-0.52	0.0032	Down
AA953_RS11200	*fabA*	-0.43	0.004	Down
AA953_RS11220	*AA953_RS11220*	-1.15	2.41E-06	Down
AA953_RS11225	*sulA*	-2.82	3.35E-149	Down
AA953_RS11265	*yccU*	-0.50	0.0092	Down
AA953_RS11485	*ymdF*	-0.78	2.84E-05	Down
AA953_RS11555	*efeO*	-1.41	4.38E-32	Down
AA953_RS11560	*efeB*	-0.73	1.32E-05	Down
AA953_RS11570	*phoH*	-0.62	0.0012	Down
AA953_RS11590	*pgaA*	-0.95	0.003	Down
AA953_RS11665	*csgD*	-1.41	0.0002	Down
AA953_RS11755	*yceA*	-0.50	0.0026	Down
AA953_RS11785	*dinI*	-2.29	8.20E-38	Down
AA953_RS11790	*pyrC*	-0.57	0.0002	Down
AA953_RS11800	*grxB*	-0.45	0.0005	Down
AA953_RS11990	*fhuE*	-4.05	3.39E-193	Down
AA953_RS12040	*bhsA*	-1.25	7.97E-05	Down
AA953_RS12180	*lit*	-0.76	8.58E-05	Down
AA953_RS12185	*intE*	-2.26	6.45E-22	Down
AA953_RS12190	*xisE*	-2.36	7.47E-11	Down
AA953_RS12195	*ymfJ*	-2.66	5.53E-50	Down
AA953_RS12205	*croE*	-3.48	1.32E-05	Down
AA953_RS12210	*ymfL*	-2.03	7.80E-07	Down
AA953_RS12215	*ymfM*	-3.28	3.97E-08	Down
AA953_RS12220	*ymfN*	-1.94	1.92E-07	Down
AA953_RS12380	*pliG*	-0.82	6.52E-05	Down
AA953_RS12385	*AA953_RS12385*	-0.80	0.0029	Down
AA953_RS12410	*umuD*	-2.92	9.42E-24	Down
AA953_RS12415	*umuC*	-1.57	1.11E-08	Down
AA953_RS12775	*yciI*	-0.83	1.10E-05	Down
AA953_RS12780	*tonB*	-1.35	3.68E-31	Down
AA953_RS12805	*yciE*	-0.89	2.31E-08	Down

AA953_RS12810	*yciF*	-0.83	6.52E-05	Down
AA953_RS12820	*trpA*	-0.69	3.48E-06	Down
AA953_RS12825	*trpB*	-0.73	6.20E-05	Down
AA953_RS12830	*trpC*	-0.52	7.09E-05	Down
AA953_RS12835	*trpD*	-0.73	2.55E-06	Down
AA953_RS12840	*trpE*	-0.81	4.81E-11	Down
AA953_RS13215	*ynaI*	-0.65	0.0003	Down
AA953_RS13460	*uspF*	-0.64	0.0004	Down
AA953_RS13505	*feaR*	-0.69	6.99E-05	Down
AA953_RS13510	*feaB*	-0.52	0.0043	Down
AA953_RS13525	*paaA*	-1.25	2.77E-10	Down
AA953_RS13545	*paaE*	-1.25	3.58E-05	Down
AA953_RS13645	*azoR*	-0.45	0.0092	Down
AA953_RS13655	*ydcF*	-0.53	0.0015	Down
AA953_RS13735	*tehA*	-1.17	8.81E-10	Down
AA953_RS13740	*tehB*	-1.18	7.18E-19	Down
AA953_RS13860	*yncD*	-0.63	0.0005	Down
AA953_RS13865	*yncE*	-2.91	3.50E-150	Down
AA953_RS13905	*pptA*	-0.57	0.0001	Down
AA953_RS13990	*adhP*	-0.43	9.84E-05	Down
AA953_RS14010	*osmC*	-0.88	3.21E-08	Down
AA953_RS14075	*pqqL*	-1.41	5.50E-40	Down
AA953_RS14080	*yddB*	-1.45	1.92E-29	Down
AA953_RS14085	*yddA*	-1.47	6.66E-15	Down
AA953_RS14260	*marR*	-1.95	1.34E-17	Down
AA953_RS14265	*marA*	-2.25	3.00E-22	Down
AA953_RS14270	*marB*	-1.49	0.001	Down
AA953_RS14295	*ydeI*	-0.66	0.0002	Down
AA953_RS14355	*nohA*	-3.56	1.21E-07	Down
AA953_RS14605	*ynfM*	-3.00	3.17E-71	Down
AA953_RS14695	*manA*	-0.37	0.0084	Down
AA953_RS14895	*gloA*	-0.42	0.0007	Down
AA953_RS15040	*sufE*	-2.78	1.96E-76	Down
AA953_RS15045	*sufS*	-2.71	2.62E-109	Down
AA953_RS15050	*sufD*	-2.73	1.77E-80	Down
AA953_RS15055	*sufC*	-2.72	4.63E-103	Down
AA953_RS15060	*sufB*	-2.72	4.92E-75	Down
AA953_RS15065	*sufA*	-3.28	3.28E-123	Down
AA953_RS15180	*ydiE*	-2.47	1.93E-36	Down
AA953_RS15270	*pfkB*	-0.48	6.00E-05	Down
AA953_RS15295	*ydjM*	-1.06	0.0098	Down
AA953_RS15365	*cho*	-1.24	5.18E-13	Down
AA953_RS15465	*gdhA*	-1.26	7.49E-24	Down
AA953_RS15605	*yeaK*	-0.42	0.0059	Down

AA953_RS15665	*yeaR*	-2.30	0.0002	Down
AA953_RS15720	*yoaA*	-0.54	0.0019	Down
AA953_RS15775	*manY*	-0.37	0.0083	Down
AA953_RS15950	*yebG*	-1.89	1.91E-21	Down
AA953_RS15970	*zwf*	-0.40	0.0073	Down
AA953_RS16015	*ruvB*	-0.77	1.21E-07	Down
AA953_RS16020	*ruvA*	-0.89	1.34E-12	Down
AA953_RS16475	*rcsA*	-2.11	1.19E-31	Down
AA953_RS16550	*hchA*	-0.57	0.0007	Down
AA953_RS16620	*shiA*	-1.32	1.58E-32	Down
AA953_RS16630	*yeeN*	-0.72	2.88E-05	Down
AA953_RS16765	*yeeX*	-0.55	0.0005	Down
AA953_RS16770	*yeeA*	-0.50	0.0094	Down
AA953_RS16775	*sbmC*	-1.37	1.01E-11	Down
AA953_RS16790	*yeeD*	-1.60	9.55E-27	Down
AA953_RS16795	*yeeE*	-1.68	1.31E-44	Down
AA953_RS16870	*ugd*	-1.97	8.98E-15	Down
AA953_RS16955	*wzc*	-1.92	4.48E-05	Down
AA953_RS16965	*wza*	-1.98	0.0013	Down
AA953_RS17155	*thiD*	-0.65	0.0011	Down
AA953_RS17170	*rcnA*	-0.86	0.0002	Down
AA953_RS17205	*yehE*	-0.85	1.92E-05	Down
AA953_RS17430	*yeiG*	-0.61	3.58E-07	Down
AA953_RS17435	*cirA*	-6.06	2.11E-274	Down
AA953_RS17455	*nfo*	-0.50	0.001	Down
AA953_RS17720	*mqo*	-1.74	6.38E-11	Down
AA953_RS17725	*yojI*	-0.58	3.33E-05	Down
AA953_RS17745	*ompC*	-1.80	1.36E-66	Down
AA953_RS17945	*nudI*	-1.27	8.35E-15	Down
AA953_RS18025	*elaB*	-0.89	3.80E-11	Down
AA953_RS18495	*yfdQ*	-1.35	0.0008	Down
AA953_RS18670	*mntH*	-1.33	4.91E-20	Down
AA953_RS18795	*ptsH*	-0.36	0.0017	Down
AA953_RS19040	*talA*	-0.62	8.09E-07	Down
AA953_RS19045	*tktB*	-0.47	0.0083	Down
AA953_RS19110	*purC*	-0.40	0.0012	Down
AA953_RS19210	*yfgD*	-0.41	0.0035	Down
AA953_RS19280	*guaB*	-0.68	8.93E-08	Down
AA953_RS19395	*iscR*	-0.93	4.22E-17	Down
AA953_RS19500	*hmp*	-3.34	3.07E-99	Down
AA953_RS19545	*yfhH*	-0.53	0.0012	Down
AA953_RS19630	*nadB*	-0.44	0.0004	Down
AA953_RS19705	*kgtP*	-0.65	1.67E-06	Down
AA953_RS19770	*tyrA*	-1.19	5.30E-13	Down

AA953_RS19775	*aroF*	-1.20	2.21E-14	Down
AA953_RS19850	*recN*	-3.19	4.17E-107	Down
AA953_RS19855	*bamE*	-0.45	0.0005	Down
AA953_RS20115	*nrdH*	-2.63	7.51E-53	Down
AA953_RS20120	*nrdI*	-2.72	9.41E-46	Down
AA953_RS20125	*nrdE*	-2.64	3.35E-78	Down
AA953_RS20130	*nrdF*	-2.53	1.06E-73	Down
AA953_RS20165	*mprA*	-0.40	0.0029	Down
AA953_RS20180	*luxS*	-0.56	6.20E-05	Down
AA953_RS20240	*recX*	-1.09	0.0004	Down
AA953_RS20245	*recA*	-1.64	2.13E-41	Down
AA953_RS20415	*ygbA*	-2.66	1.40E-21	Down
AA953_RS20420	*mutS*	-0.38	0.0016	Down
AA953_RS20570	*cysH*	-0.46	0.0026	Down
AA953_RS20655	*eno*	-0.85	6.31E-10	Down
AA953_RS20810	*ygdI*	-0.80	9.05E-05	Down
AA953_RS20855	*argA*	-0.45	0.0011	Down
AA953_RS21275	*yqfA*	-1.24	2.84E-11	Down
AA953_RS21395	*mscS*	-0.50	0.0012	Down
AA953_RS21665	*glcF*	-0.50	0.0061	Down
AA953_RS21840	*exbD*	-1.73	1.41E-47	Down
AA953_RS21845	*exbB*	-1.90	9.83E-54	Down
AA953_RS21955	*mdaB*	-1.07	1.61E-10	Down
AA953_RS22030	*yqiC*	-0.49	7.80E-05	Down
AA953_RS22185	*yqjH*	-2.27	8.81E-71	Down
AA953_RS22190	*yqjI*	-1.05	1.46E-09	Down
AA953_RS22620	*yhbW*	-0.80	1.69E-07	Down
AA953_RS22860	*yrbL*	-0.62	2.93E-05	Down
AA953_RS23000	*mdh*	-0.62	2.71E-06	Down
AA953_RS23005	*argR*	-0.78	4.16E-12	Down
AA953_RS00315	*bfr*	2.02	3.70E-70	Up
AA953_RS00470	*nirB*	3.51	0.0004	Up
AA953_RS01135	*dtpB*	0.61	0.003	Up
AA953_RS01250	*gadW*	0.75	0.0006	Up
AA953_RS01300	*yhjH*	3.49	5.31E-08	Up
AA953_RS01340	*bcsB*	0.39	0.007	Up
AA953_RS01345	*bcsA*	0.49	0.001	Up
AA953_RS01390	*dppF*	1.84	1.01E-44	Up
AA953_RS01395	*dppD*	1.57	4.92E-28	Up
AA953_RS01400	*dppC*	1.81	1.79E-19	Up
AA953_RS01405	*dppB*	1.47	6.81E-08	Up
AA953_RS01430	*eptB*	0.48	0.0025	Up
AA953_RS01560	*bax*	0.58	0.001	Up
AA953_RS01565	*malS*	1.21	4.05E-08	Up

AA953_RS01765	*cysE*	0.81	4.01E-10	Up
AA953_RS02130	*yidF*	0.66	0.0052	Up
AA953_RS02310	*tnaA*	0.54	0.0027	Up
AA953_RS02630	*ilvA*	0.42	0.0092	Up
AA953_RS03100	*hemN*	0.77	1.07E-11	Up
AA953_RS03120	*glnA*	0.44	2.62E-05	Up
AA953_RS03250	*fdoG*	0.50	2.45E-05	Up
AA953_RS03420	*glpK*	0.66	0.0007	Up
AA953_RS03505	*katG*	0.91	2.06E-07	Up
AA953_RS03575	*ppc*	0.39	0.0048	Up
AA953_RS03885	*aceB*	1.15	3.94E-21	Up
AA953_RS03890	*aceA*	1.32	4.81E-21	Up
AA953_RS03895	*aceK*	1.26	3.09E-10	Up
AA953_RS03910	*metH*	0.46	0.0003	Up
AA953_RS03915	*yjbB*	0.71	3.58E-06	Up
AA953_RS04085	*pspG*	1.69	5.98E-16	Up
AA953_RS04230	*yjcO*	0.47	2.93E-05	Up
AA953_RS04290	*alsB*	0.70	0.002	Up
AA953_RS04560	*aspA*	0.44	0.0025	Up
AA953_RS04630	*frdD*	1.80	2.57E-09	Up
AA953_RS04635	*frdC*	1.37	4.48E-06	Up
AA953_RS04640	*frdB*	1.07	5.23E-07	Up
AA953_RS04645	*frdA*	1.17	4.40E-15	Up
AA953_RS04945	*cpdB*	0.46	0.0007	Up
AA953_RS04960	*ytfJ*	0.45	0.0091	Up
AA953_RS05535	*uxuA*	1.20	2.90E-15	Up
AA953_RS05540	*uxuB*	0.88	3.65E-09	Up
AA953_RS05720	*tsr*	1.23	1.43E-06	Up
AA953_RS05730	*lgoR*	0.90	4.14E-05	Up
AA953_RS05970	*arcA*	0.42	0.0003	Up
AA953_RS06115	*ispH*	0.54	0.001	Up
AA953_RS06575	*acnB*	0.73	0.0008	Up
AA953_RS07625	*yahN*	0.83	0.0011	Up
AA953_RS07640	*prpB*	2.07	1.10E-18	Up
AA953_RS07645	*prpC*	1.84	8.75E-16	Up
AA953_RS07650	*prpD*	2.26	5.30E-19	Up
AA953_RS07655	*prpE*	1.80	4.81E-09	Up
AA953_RS08050	*tsx*	0.98	7.37E-14	Up
AA953_RS08095	*dxs*	0.67	9.84E-07	Up
AA953_RS08765	*nmpC*	1.56	2.09E-27	Up
AA953_RS09020	*cstA*	0.48	0.0071	Up
AA953_RS09145	*pagP(crcA)*	1.26	0.0006	Up
AA953_RS09305	*gltJ*	0.58	0.0036	Up
AA953_RS09335	*miaB*	0.45	0.0009	Up

AA953_RS09425	*fur*	9.90	4.96E-77	Up
AA953_RS09650	*sdhA*	0.39	0.0012	Up
AA953_RS09655	*sdhB*	0.71	1.28E-08	Up
AA953_RS09865	*modA*	3.00	7.28E-166	Up
AA953_RS09870	*modB*	1.71	2.93E-11	Up
AA953_RS09875	*modC*	1.36	6.44E-11	Up
AA953_RS10485	*mntS*	2.68	0.0032	Up
AA953_RS10890	*serS*	0.30	0.0086	Up
AA953_RS10895	*dmsA*	1.23	0.0009	Up
AA953_RS10900	*dmsB*	1.97	0.0063	Up
AA953_RS10935	*pflA*	0.65	0.0005	Up
AA953_RS11385	*insB*	1.08	0.0012	Up
AA953_RS11830	*flgN*	1.61	2.65E-05	Up
AA953_RS11835	*flgM*	1.49	4.23E-05	Up
AA953_RS11840	*flgA*	1.60	0.0003	Up
AA953_RS11845	*flgB*	3.62	1.04E-19	Up
AA953_RS11850	*flgC*	5.57	7.62E-12	Up
AA953_RS11855	*flgD*	4.22	3.64E-36	Up
AA953_RS11860	*flgE*	3.25	1.04E-39	Up
AA953_RS11865	*flgF*	2.94	1.14E-09	Up
AA953_RS11870	*flgG*	2.68	8.54E-22	Up
AA953_RS11875	*flgH*	2.11	2.82E-05	Up
AA953_RS11880	*flgI*	2.95	4.16E-14	Up
AA953_RS11885	*flgJ*	2.10	8.84E-09	Up
AA953_RS11890	*flgK*	2.05	1.59E-20	Up
AA953_RS11895	*flgL*	1.76	3.80E-23	Up
AA953_RS12465	*ycgR*	1.57	1.91E-09	Up
AA953_RS12730	*oppA*	0.49	1.15E-05	Up
AA953_RS12735	*oppB*	1.18	4.95E-06	Up
AA953_RS12740	*oppC*	1.03	1.50E-06	Up
AA953_RS12745	*oppD*	0.93	3.58E-07	Up
AA953_RS12750	*oppF*	0.96	2.34E-07	Up
AA953_RS12920	*acnA*	0.80	2.03E-06	Up
AA953_RS13085	*pspC*	2.01	1.34E-12	Up
AA953_RS13580	*paaX*	0.48	0.0097	Up
AA953_RS13585	*paaY*	0.64	5.97E-05	Up
AA953_RS13692	*mokB*	0.85	0.0002	Up
AA953_RS13695	*trg*	0.74	0.006	Up
AA953_RS13745	*ydcL*	0.73	1.84E-08	Up
AA953_RS13800	*ydcS*	0.43	0.008	Up
AA953_RS13870	*ansP*	0.68	0.0016	Up
AA953_RS13935	*narY*	1.03	0.0045	Up
AA953_RS13940	*narZ*	1.19	2.51E-09	Up
AA953_RS13945	*narU*	0.88	0.0043	Up

AA953_RS14035	*ddpA*	0.52	0.0006	Up
AA953_RS14450	*flxA*	1.28	0.009	Up
AA953_RS14530	*rspA*	0.99	2.69E-10	Up
AA953_RS14615	*ydgU*	2.08	0.0083	Up
AA953_RS14635	*tqsA*	1.13	2.41E-06	Up
AA953_RS14685	*fumC*	0.92	1.85E-07	Up
AA953_RS14690	*fumA*	1.64	6.62E-60	Up
AA953_RS14745	*add*	0.72	0.0002	Up
AA953_RS14810	*dtpA*	0.59	0.0031	Up
AA953_RS14910	*grxD*	0.74	1.30E-07	Up
AA953_RS14920	*sodB*	3.90	2.21E-128	Up
AA953_RS15075	*menI*	0.59	0.0037	Up
AA953_RS15445	*ynjE*	0.55	0.0022	Up
AA953_RS15690	*yeaW*	1.64	0.0014	Up
AA953_RS15995	*mepM*	0.36	0.0048	Up
AA953_RS16110	*flhA*	1.11	4.49E-05	Up
AA953_RS16115	*flhB*	2.14	1.39E-06	Up
AA953_RS16135	*cheR*	2.09	1.65E-05	Up
AA953_RS16140	*tap*	3.08	4.33E-17	Up
AA953_RS16145	*tar*	2.80	1.59E-18	Up
AA953_RS16150	*cheW*	2.28	2.50E-13	Up
AA953_RS16155	*cheA*	2.84	3.11E-29	Up
AA953_RS16160	*motB*	2.08	8.82E-05	Up
AA953_RS16165	*motA*	3.69	1.23E-15	Up
AA953_RS16170	*flhC*	0.77	2.02E-07	Up
AA953_RS16215	*araG*	0.99	0.0053	Up
AA953_RS16220	*araF*	0.99	7.29E-05	Up
AA953_RS16225	*ftnB*	0.87	3.35E-10	Up
AA953_RS16240	*yecR*	2.86	1.57E-05	Up
AA953_RS16245	*ftnA*	0.68	0.0002	Up
AA953_RS16305	*sdiA*	0.72	1.48E-05	Up
AA953_RS16335	*fliA*	3.38	2.51E-29	Up
AA953_RS16340	*fliC*	3.23	3.42E-95	Up
AA953_RS16345	*fliD*	2.78	2.55E-12	Up
AA953_RS16370	*yedE*	0.55	0.0007	Up
AA953_RS16405	*fliE*	2.27	0.0001	Up
AA953_RS16410	*fliF*	3.71	1.03E-36	Up
AA953_RS16415	*fliG*	3.49	9.50E-24	Up
AA953_RS16420	*fliH*	2.91	6.68E-09	Up
AA953_RS16425	*fliI*	3.10	2.62E-15	Up
AA953_RS16435	*fliK*	2.66	3.17E-09	Up
AA953_RS16440	*fliL*	3.58	4.98E-13	Up
AA953_RS16445	*fliM*	2.89	9.01E-12	Up
AA953_RS16450	*fliN*	2.85	0.0014	Up

AA953_RS16460	*fliP*	2.18	0.0002	Up
AA953_RS17210	*mrp*	0.75	1.11E-08	Up
AA953_RS17365	*cdd*	0.52	0.009	Up
AA953_RS17380	*preT*	2.34	7.61E-15	Up
AA953_RS17385	*preA*	2.00	1.39E-10	Up
AA953_RS17400	*mglB*	0.54	0.0043	Up
AA953_RS17715	*eco*	0.92	1.28E-05	Up
AA953_RS17885	*glpT*	1.00	2.14E-05	Up
AA953_RS18075	*nuoN*	0.62	8.84E-06	Up
AA953_RS18080	*nuoM*	0.52	0.0006	Up
AA953_RS18085	*nuoL*	0.68	0.0012	Up
AA953_RS18100	*nuoI*	0.86	6.47E-06	Up
AA953_RS18105	*nuoH*	0.83	3.04E-07	Up
AA953_RS18110	*nuoG*	0.79	7.31E-08	Up
AA953_RS18115	*nuoF*	0.61	0.0001	Up
AA953_RS18125	*nuoC*	0.92	8.63E-08	Up
AA953_RS18130	*nuoB*	0.88	1.95E-07	Up
AA953_RS18135	*nuoA*	0.86	5.54E-14	Up
AA953_RS18140	*lrhA*	0.65	7.37E-06	Up
AA953_RS18415	*fadL*	0.94	6.54E-14	Up
AA953_RS18640	*fryC*	3.00	0.0092	Up
AA953_RS18850	*ucpA*	0.45	0.0027	Up
AA953_RS19360	*iscX*	0.95	0.0023	Up
AA953_RS19370	*hscA*	0.39	0.004	Up
AA953_RS19390	*iscS*	0.44	0.0031	Up
AA953_RS20135	*proV*	0.54	0.0004	Up
AA953_RS21120	*ygeV*	0.50	5.10E-05	Up
AA953_RS21155	*yqeC*	0.97	0.0017	Up
AA953_RS21160	*mocA*	0.85	0.0006	Up
AA953_RS21260	*cptA*	0.87	0.0001	Up
AA953_RS21450	*loiP*	0.80	3.01E-07	Up
AA953_RS21625	*yghF*	2.55	0.0003	Up
AA953_RS21635	*pppA*	0.98	0.0039	Up
AA953_RS21640	*sslE*	1.51	1.09E-23	Up
AA953_RS21795	*hybB*	2.42	0.0014	Up
AA953_RS21805	*hybO*	1.44	2.93E-05	Up
AA953_RS21815	*yghW*	2.27	0.0051	Up
AA953_RS21895	*ygiQ*	0.81	3.23E-10	Up
AA953_RS22240	*fadH*	0.90	1.31E-14	Up
AA953_RS22260	*rlmG*	0.63	0.0023	Up
AA953_RS22280	*alx*	1.27	4.43E-05	Up
AA953_RS22295	*uxaA*	0.94	3.58E-08	Up
AA953_RS22300	*uxaC*	0.80	0.0004	Up
AA953_RS22305	*exuT*	1.01	1.71E-06	Up

AA953_RS22635	*deaD*	0.51	0.0023	Up
AA953_RS22890	*gltD*	0.79	9.40E-16	Up
AA953_RS23105	*accB*	0.47	0.0008	Up
AA953_RS23175	*yhdW*	2.02	9.69E-18	Up
AA953_RS23325	*traW*	1.31	0.0043	Up
AA953_RS23335	*trbC*	1.56	0.005	Up
AA953_RS23405	*traD*	0.83	0.0011	Up
AA953_RS23410	*orf5*	0.59	0.0004	Up
AA953_RS23420	*traI*	0.56	0.0004	Up
AA953_RS23475	*AA953_RS23475*	0.52	0.0025	Up
AA953_RS23490	*yebA*	0.66	0.0015	Up
AA953_RS23495	*yebB(F)*	0.70	1.28E-05	Up
AA953_RS23500	*pifA*	0.52	0.0004	Up

Table S3. DEGs identified comparing the transcriptomes of NCM3722 (Fur+) versus that of CY405 (Fur-) grown in the medium without iron supplied.

Gene ID	Gene Symbol	Log_2 FC (Fur+ vs Fur-)	*q-value*	Diff
AA953_RS01940	*pyrE*	-0.72	0.0023	Down
AA953_RS02115	*AA953_RS02115*	-1.71	0.0071	Down
AA953_RS02120	*tisB*	-2.23	0.0069	Down
AA953_RS02640	*ilvC*	-0.78	0.0023	Down
AA953_RS03385	*tpiA*	-0.86	0.0012	Down
AA953_RS03940	*pgi*	-0.58	0.0006	Down
AA953_RS04385	*yjdM*	-0.80	0.0002	Down
AA953_RS05315	*fecC*	-1.04	0.0097	Down
AA953_RS05325	*fecA*	-1.16	0.0065	Down
AA953_RS05775	*bglJ*	-0.82	0.0008	Down
AA953_RS05785	*yjjZ*	-4.26	3.46E-10	Down
AA953_RS06090	*yaaY*	-0.90	0.0018	Down
AA953_RS06125	*dapB*	-1.08	0.0009	Down
AA953_RS07025	*dkgB*	-1.44	3.94E-06	Down
AA953_RS07100	*ivy*	-0.64	0.0068	Down
AA953_RS07190	*gpt*	-0.96	0.0002	Down
AA953_RS08125	*yajQ*	-0.76	0.0078	Down
AA953_RS08420	*copA*	-0.97	0.0009	Down
AA953_RS08810	*nohD2*	-1.59	0.0077	Down
AA953_RS08860	*nfrB*	-1.22	0.0047	Down
AA953_RS08995	*entC*	-1.46	0.0086	Down
AA953_RS09630	*gltA*	-0.93	0.0019	Down
AA953_RS09635	*AA953_RS09635*	-0.86	0.0038	Down
AA953_RS09825	*gpmA*	-2.02	1.06E-22	Down
AA953_RS09880	*ybhA*	-0.74	0.0006	Down
AA953_RS10095	*AA953_RS10095*	-1.94	0.0014	Down
AA953_RS10100	*AA953_RS10100*	-1.82	0.0047	Down
AA953_RS10105	*rzpD2*	-1.27	0.0023	Down
AA953_RS10165	*AA953_RS10165*	-2.68	2.99E-05	Down
AA953_RS10190	*AA953_RS10190*	-1.84	0.0047	Down
AA953_RS10195	*AA953_RS10195*	-2.43	0.0002	Down
AA953_RS10210	*AA953_RS10210*	-2.12	0.0004	Down
AA953_RS10215	*AA953_RS10215*	-2.11	0.005	Down
AA953_RS10240	*tfaQ2*	-2.17	0.0078	Down
AA953_RS10255	*AA953_RS10255*	-0.64	0.0038	Down
AA953_RS10265	*bioA*	-1.25	1.04E-11	Down
AA953_RS10275	*bioF*	-0.57	0.0042	Down
AA953_RS10300	*AA953_RS10300*	-1.72	0.0062	Down
AA953_RS10305	*moaA*	-1.82	1.42E-11	Down
AA953_RS10310	*moaB*	-2.06	4.04E-11	Down

AA953_RS10315	*moaC*	-2.09	2.17E-13	Down
AA953_RS10325	*moaE*	-2.36	5.37E-07	Down
AA953_RS12410	*umuD*	-1.90	0.0015	Down
AA953_RS12835	*trpD*	-0.78	0.0097	Down
AA953_RS13525	*paaA*	-0.95	0.0019	Down
AA953_RS13740	*tehB*	-0.78	0.0007	Down
AA953_RS13865	*yncE*	-1.15	0.0075	Down
AA953_RS14095	*ydeN*	-1.15	0.0062	Down
AA953_RS14260	*marR*	-1.71	0.0002	Down
AA953_RS14265	*marA*	-1.56	0.0005	Down
AA953_RS14605	*ynfM*	-2.82	1.13E-14	Down
AA953_RS15040	*sufE*	-1.27	6.76E-06	Down
AA953_RS15065	*sufA*	-1.41	1.06E-06	Down
AA953_RS15180	*ydiE*	-1.21	0.0027	Down
AA953_RS15365	*cho*	-0.87	0.0025	Down
AA953_RS15465	*gdhA*	-1.12	9.87E-05	Down
AA953_RS16475	*rcsA*	-1.75	2.38E-06	Down
AA953_RS16510	*yedA*	-1.44	0.005	Down
AA953_RS16630	*yeeN*	-0.87	7.69E-05	Down
AA953_RS16790	*yeeD*	-1.51	1.89E-07	Down
AA953_RS16795	*yeeE*	-1.47	7.25E-17	Down
AA953_RS16965	*wza*	-2.43	0.0006	Down
AA953_RS17440	*lysP*	-0.64	0.0097	Down
AA953_RS17720	*mqo*	-1.22	4.49E-05	Down
AA953_RS17745	*ompC*	-1.72	3.16E-13	Down
AA953_RS17945	*nudI*	-1.31	0.0004	Down
AA953_RS18670	*mntH*	-2.39	0.0043	Down
AA953_RS19110	*purC*	-0.50	0.0053	Down
AA953_RS19500	*hmp*	-2.39	1.24E-08	Down
AA953_RS19770	*tyrA*	-1.37	9.06E-13	Down
AA953_RS19775	*aroF*	-1.45	1.03E-12	Down
AA953_RS20415	*ygbA*	-2.27	2.99E-05	Down
AA953_RS20655	*eno*	-0.83	1.38E-06	Down
AA953_RS21275	*yqfA*	-1.33	3.20E-05	Down
AA953_RS21840	*exbD*	-0.92	0.0069	Down
AA953_RS21845	*exbB*	-0.85	0.0097	Down
AA953_RS21955	*mdaB*	-0.90	0.0014	Down
AA953_RS22185	*yqjH*	-1.29	6.00E-07	Down
AA953_RS23580	*AA953_RS23580*	-2.59	0.0077	Down
AA953_RS00315	*bfr*	1.08	0.0091	Up
AA953_RS01300	*yhjH*	3.28	1.53E-08	Up
AA953_RS01390	*dppF*	1.49	1.18E-11	Up
AA953_RS01395	*dppD*	1.36	3.46E-10	Up
AA953_RS01400	*dppC*	1.48	4.19E-06	Up

AA953_RS01405	*dppB*	1.36	0.0079	Up
AA953_RS01765	*cysE*	0.84	1.24E-07	Up
AA953_RS03100	*hemN*	0.57	0.0079	Up
AA953_RS03115	*glnL*	0.66	0.0076	Up
AA953_RS03885	*aceB*	1.09	0.0002	Up
AA953_RS03890	*aceA*	1.21	2.64E-05	Up
AA953_RS03895	*aceK*	1.07	0.0005	Up
AA953_RS04395	*yjcZ*	1.54	0.0015	Up
AA953_RS04630	*frdD*	1.41	0.0012	Up
AA953_RS04635	*frdC*	1.04	0.0068	Up
AA953_RS04640	*frdB*	1.01	0.0004	Up
AA953_RS04645	*frdA*	1.16	7.78E-07	Up
AA953_RS05720	*tsr*	1.49	1.36E-06	Up
AA953_RS07640	*prpB*	2.45	1.66E-12	Up
AA953_RS07645	*prpC*	2.59	1.03E-12	Up
AA953_RS07650	*prpD*	2.67	0.0024	Up
AA953_RS07655	*prpE*	1.68	0.0012	Up
AA953_RS07920	*proC*	0.83	0.0008	Up
AA953_RS08050	*tsx*	0.69	0.0003	Up
AA953_RS08765	*nmpC*	0.88	1.04E-06	Up
AA953_RS09425	*fur*	9.76	4.04E-65	Up
AA953_RS09865	*modA*	4.23	2.13E-84	Up
AA953_RS09870	*modB*	3.47	4.43E-24	Up
AA953_RS09875	*modC*	2.44	1.36E-18	Up
AA953_RS10895	*dmsA*	1.29	0.0038	Up
AA953_RS11830	*flgN*	1.39	0.0038	Up
AA953_RS11835	*flgM*	1.18	0.0022	Up
AA953_RS11850	*flgC*	2.94	1.64E-09	Up
AA953_RS11855	*flgD*	2.81	2.31E-09	Up
AA953_RS11860	*flgE*	2.64	0.0018	Up
AA953_RS11865	*flgF*	2.38	0.0004	Up
AA953_RS11870	*flgG*	2.35	1.69E-09	Up
AA953_RS11875	*flgH*	2.89	1.10E-09	Up
AA953_RS11880	*flgI*	2.27	6.96E-09	Up
AA953_RS11885	*flgJ*	2.14	3.10E-08	Up
AA953_RS11890	*flgK*	2.14	2.20E-14	Up
AA953_RS11895	*flgL*	1.60	3.45E-14	Up
AA953_RS12465	*ycgR*	1.47	3.31E-05	Up
AA953_RS12740	*oppC*	1.10	0.0091	Up
AA953_RS12750	*oppF*	1.12	0.0011	Up
AA953_RS13695	*trg*	0.89	0.0074	Up
AA953_RS13935	*narY*	1.19	0.0018	Up
AA953_RS13940	*narZ*	1.32	0.0003	Up
AA953_RS14525	*rspB*	0.98	0.0091	Up

AA953_RS14530	*rspA*	0.95	6.77E-05	Up
AA953_RS14565	*ynfF*	1.08	0.0054	Up
AA953_RS14690	*fumA*	1.45	1.89E-07	Up
AA953_RS16110	*flhA*	1.01	0.0068	Up
AA953_RS16115	*flhB*	1.83	0.0042	Up
AA953_RS16135	*cheR*	1.64	0.0078	Up
AA953_RS16140	*tap*	2.97	1.15E-13	Up
AA953_RS16145	*tar*	2.73	1.25E-14	Up
AA953_RS16150	*cheW*	2.00	1.24E-09	Up
AA953_RS16155	*cheA*	2.25	6.76E-14	Up
AA953_RS16160	*motB*	2.12	3.67E-05	Up
AA953_RS16165	*motA*	2.64	1.23E-12	Up
AA953_RS16240	*yecR*	1.85	0.0024	Up
AA953_RS16330	*fliZ*	1.97	1.36E-06	Up
AA953_RS16335	*fliA*	2.82	1.50E-08	Up
AA953_RS16340	*fliC*	2.92	1.17E-33	Up
AA953_RS16345	*fliD*	3.01	1.15E-10	Up
AA953_RS16350	*fliS*	2.74	0.0009	Up
AA953_RS16405	*fliE*	2.06	0.0005	Up
AA953_RS16410	*fliF*	2.92	1.92E-14	Up
AA953_RS16415	*fliG*	2.13	3.36E-08	Up
AA953_RS16420	*fliH*	2.33	1.84E-06	Up
AA953_RS16425	*fliI*	3.06	1.93E-10	Up
AA953_RS16435	*fliK*	2.26	1.23E-06	Up
AA953_RS16440	*fliL*	2.45	1.29E-07	Up
AA953_RS16445	*fliM*	2.53	2.66E-08	Up
AA953_RS16450	*fliN*	3.60	0.0023	Up
AA953_RS17380	*preT*	1.45	0.0018	Up
AA953_RS17715	*eco*	1.91	2.37E-13	Up
AA953_RS18105	*nuoH*	1.03	0.0019	Up
AA953_RS18110	*nuoG*	0.98	8.65E-05	Up
AA953_RS18125	*nuoC*	1.04	0.0012	Up
AA953_RS18130	*nuoB*	0.93	0.0001	Up
AA953_RS19350	*sseB*	0.70	0.0008	Up
AA953_RS19365	*fdx*	0.75	0.0052	Up
AA953_RS19370	*hscA*	0.59	0.0014	Up
AA953_RS19385	*iscU*	0.92	0.002	Up
AA953_RS19390	*iscS*	1.09	0.0004	Up
AA953_RS21105	*xdhA*	0.71	0.0062	Up
AA953_RS21155	*yqeC*	1.10	0.0097	Up
AA953_RS21625	*yghF*	2.19	0.0047	Up
AA953_RS21640	*sslE*	1.37	3.00E-09	Up
AA953_RS21815	*yghW*	4.64	0.0001	Up
AA953_RS22240	*fadH*	0.97	8.35E-06	Up

AA953_RS22890	*gltD*	0.67	0.0069	Up
AA953_RS23175	*yhdW*	1.51	4.11E-06	Up

Table S4. Binding peaks of Fur identified in this work.

Peak postion[a]	Peak fold enrichment	Gene ID	Gene symbol
Up stream	1.11	AA953_RS00005	*rrfD*
Up stream	1.19	AA953_RS00045	*tsaC*
Up stream	1.33	AA953_RS00080	*trkA*
Up stream	1.29	AA953_RS00095	*zntR*
Up stream	1.24	AA953_RS00100	*yhdN*
Up stream	1.38	AA953_RS00120	*rpsK*
Up stream	1.30	AA953_RS00125	*rpsM*
Up stream	1.27	AA953_RS00155	*rplR*
Up stream	1.43	AA953_RS00170	*rpsN*
Up stream	1.55	AA953_RS00195	*rpmC*
Up stream	1.23	AA953_RS00240	*rpsJ*
Up stream	2.51	AA953_RS00320	*bfd*
Up stream	1.28	AA953_RS00500	*frlB*
Up stream	1.17	AA953_RS00585	*aroB*
Up stream	1.26	AA953_RS00675	*greB*
Up stream	6.98	AA953_RS00685	*feoA*
Up stream	1.14	AA953_RS00770	*glpD*
Up stream	1.37	AA953_RS00805	*asd*
Up stream	50.03	AA953_RS00840	*yhhY*
Up stream	1.30	AA953_RS00890	*ugpE*
Up stream	1.41	AA953_RS00900	*ugpB*
Up stream	2.16	AA953_RS00935	*panM*
Up stream	1.17	AA953_RS00945	*livJ*
Up stream	1.31	AA953_RS00950	*rpoH*
Up stream	1.20	AA953_RS01075	*yhhJ*
Up stream	1.31	AA953_RS01255	*gadX*
Up stream	1.27	AA953_RS01500	*glyS*
Up stream	2.17	AA953_RS01550	*xylH*
Up stream	1.16	AA953_RS01825	*rfaD*
Up stream	1.10	AA953_RS01830	*waaF*
Up stream	1.38	AA953_RS02120	*tisB*
Up stream	1.42	AA953_RS02125	*emrD*
Up stream	1.30	AA953_RS02215	*dgoT*
Up stream	1.22	AA953_RS02250	*yidB*
Up stream	1.15	AA953_RS02320	*mdtL*
Up stream	1.18	AA953_RS02435	*atpG*
Up stream	1.27	AA953_RS02450	*atpF*
Up stream	1.09	AA953_RS02565	*gltU*
Up stream	1.12	AA953_RS02575	*rrfC*
Up stream	1.37	AA953_RS02610	*ilvG*
Up stream	1.24	AA953_RS02645	*ppiC*

Up stream	1.11	AA953_RS02660	*gpp*
Up stream	1.27	AA953_RS02935	*rmuC*
Up stream	1.16	AA953_RS02965	*tatC*
Up stream	1.08	AA953_RS03040	*rrfA*
Up stream	1.13	AA953_RS03065	*dsbA*
Up stream	1.32	AA953_RS03085	*polA*
Up stream	1.95	AA953_RS03115	*glnL*
Up stream	1.86	AA953_RS03200	*yihX*
Up stream	1.62	AA953_RS03235	*fdhE*
Up stream	12.36	AA953_RS03330	*sodA*
Up stream	1.48	AA953_RS03395	*yiiR*
Up stream	1.15	AA953_RS03400	*yiiS*
Up stream	1.34	AA953_RS03430	*zapB*
Up stream	1.32	AA953_RS03570	*eptC*
Up stream	1.34	AA953_RS03605	*oxyR*
Up stream	1.08	AA953_RS03645	*gltT*
Up stream	1.14	AA953_RS03655	*rrfB*
Up stream	1.36	AA953_RS03655	*rrfB*
Up stream	1.33	AA953_RS03685	*tyrU*
Up stream	1.18	AA953_RS03705	*secE*
Up stream	1.37	AA953_RS03730	*rplL*
Up stream	1.22	AA953_RS03745	*yjaZ*
Up stream	1.16	AA953_RS03855	*gltV*
Up stream	1.35	AA953_RS04060	*yjbJ*
Up stream	2.20	AA953_RS04170	*yjcF*
Up stream	1.87	AA953_RS04530	*cadC*
Up stream	1.20	AA953_RS04540	*yjdC*
Up stream	1.19	AA953_RS04590	*yjeJ*
Up stream	1.17	AA953_RS04755	*yjeT*
Up stream	2.28	AA953_RS05070	*treC*
Up stream	1.28	AA953_RS05120	*bdcA*
Up stream	1.18	AA953_RS05185	*lptG*
Up stream	1.39	AA953_RS05265	*yjhC*
Up stream	6.00	AA953_RS05325	*fecA*
Up stream	12.35	AA953_RS05335	*fecI*
Up stream	2.93	AA953_RS05380	*yjhI*
Up stream	1.33	AA953_RS05430	*yjhX*
Up stream	15.22	AA953_RS05655	*yjiT*
Up stream	1.50	AA953_RS05775	*bglJ*
Up stream	56.70	AA953_RS05790	*leuV*
Up stream	1.32	AA953_RS05850	*yjjW*
Up stream	1.21	AA953_RS06025	*talB*
Up stream	1.16	AA953_RS06125	*dapB*
Up stream	1.27	AA953_RS06260	*djlA*

Up stream	1.45	AA953_RS06300	*araC*
Up stream	1.21	AA953_RS06340	*leuD*
Up stream	1.44	AA953_RS06390	*mraZ*
Up stream	1.11	AA953_RS06440	*murC*
Up stream	1.42	AA953_RS06445	*ddl*
Up stream	1.54	AA953_RS06465	*lpxC*
Up stream	1.17	AA953_RS06470	*secM*
Up stream	2.29	AA953_RS06480	*mutT*
Up stream	1.16	AA953_RS06495	*zapD*
Up stream	1.29	AA953_RS06580	*yacL*
Up stream	1.22	AA953_RS06625	*yadH*
Up stream	2.11	AA953_RS06640	*panD*
Up stream	1.71	AA953_RS06645	*yadD*
Up stream	1.37	AA953_RS06700	*pcnB*
Up stream	1.20	AA953_RS06735	*mrcB*
Up stream	33.06	AA953_RS06740	*fhuA*
Up stream	1.64	AA953_RS06745	*fhuC*
Up stream	1.22	AA953_RS06765	*clcA*
Up stream	1.33	AA953_RS06795	*degP*
Up stream	2.79	AA953_RS06825	*map*
Up stream	3.77	AA953_RS06830	*rpsB*
Up stream	1.18	AA953_RS06880	*lpxD*
Up stream	1.20	AA953_RS06885	*fabZ*
Up stream	1.28	AA953_RS06965	*tsaA*
Up stream	1.23	AA953_RS06970	*rcsF*
Up stream	1.10	AA953_RS07000	*ileV*
Up stream	1.16	AA953_RS07015	*rrfH*
Up stream	1.87	AA953_RS07145	*lfhA*
Up stream	1.34	AA953_RS07155	*dinB*
Up stream	1.35	AA953_RS07170	*yafP*
Up stream	1.98	AA953_RS07185	*pepD*
Up stream	1.19	AA953_RS07235	*ykfG*
Up stream	1.20	AA953_RS07305	*ykfC*
Up stream	2.12	AA953_RS07375	*intF*
Up stream	1.16	AA953_RS07610	*yahK*
Up stream	1.83	AA953_RS07620	*yahM*
Up stream	1.70	AA953_RS07800	*yaiS*
Up stream	1.33	AA953_RS08050	*tsx*
Up stream	1.13	AA953_RS08070	*ribH*
Up stream	1.56	AA953_RS08185	*clpP*
Up stream	1.32	AA953_RS08190	*clpX*
Up stream	1.36	AA953_RS08195	*lon*
Up stream	1.20	AA953_RS08265	*ybaY*
Up stream	6.99	AA953_RS08340	*ybaN*

Up stream	4.50	AA953_RS08370	*adk*
Up stream	1.25	AA953_RS08600	*purK*
Up stream	1.19	AA953_RS08605	*purE*
Up stream	2.43	AA953_RS08660	*sfmF*
Up stream	1.19	AA953_RS08735	*ybcN*
Up stream	1.19	AA953_RS08750	*rusA*
Up stream	1.23	AA953_RS08775	*rrrD*
Up stream	1.16	AA953_RS08940	*entD*
Up stream	46.07	AA953_RS08950	*fes*
Up stream	1.18	AA953_RS08965	*fepE*
Up stream	46.81	AA953_RS08990	*fepB*
Up stream	1.18	AA953_RS09005	*entB*
Up stream	1.22	AA953_RS09015	*entH*
Up stream	1.51	AA953_RS09025	*ybdD*
Up stream	1.23	AA953_RS09120	*citD*
Up stream	1.16	AA953_RS09160	*ybeM*
Up stream	1.21	AA953_RS09170	*lipA*
Up stream	1.25	AA953_RS09220	*rsfS*
Up stream	1.20	AA953_RS09230	*nadD*
Up stream	1.24	AA953_RS09265	*djlB*
Up stream	1.11	AA953_RS09290	*rihA*
Up stream	1.40	AA953_RS09300	*gltK*
Up stream	1.24	AA953_RS09310	*gltI*
Up stream	1.32	AA953_RS09325	*ybeY*
Up stream	1.46	AA953_RS09390	*nagC*
Up stream	1.18	AA953_RS09415	*chiP*
Up stream	1.28	AA953_RS09420	*chiQ(ybfN)*
Up stream	1.24	AA953_RS09460	*pgm*
Up stream	1.23	AA953_RS09465	*ybfP*
Up stream	1.13	AA953_RS09480	*potE*
Up stream	1.22	AA953_RS09570	*phr*
Up stream	1.88	AA953_RS09600	*nei*
Up stream	1.13	AA953_RS09625	*ybgD*
Up stream	1.19	AA953_RS09650	*sdhA*
Up stream	2.20	AA953_RS09655	*sdhB*
Up stream	1.15	AA953_RS09670	*sucB*
Up stream	1.24	AA953_RS09675	*sucC*
Up stream	1.21	AA953_RS09735	*tolR*
Up stream	1.32	AA953_RS09745	*tolB*
Up stream	1.35	AA953_RS09750	*pal*
Up stream	1.26	AA953_RS09755	*ybgF*
Up stream	1.17	AA953_RS09760	*lysT*
Up stream	1.20	AA953_RS09800	*pnuC*
Up stream	14.73	AA953_RS09825	*gpmA*

Up stream	1.63	AA953_RS09835	*galK*
Up stream	1.13	AA953_RS09935	*AA953_RS09935*
Up stream	1.12	AA953_RS09940	*TraR*
Up stream	1.12	AA953_RS09970	*AA953_RS09970*
Up stream	1.35	AA953_RS10030	*AA953_RS10030*
Up stream	1.24	AA953_RS10145	*parB*
Up stream	1.50	AA953_RS10245	*AA953_RS10245*
Up stream	1.14	AA953_RS10280	*bioC*
Up stream	1.21	AA953_RS10295	*ybhK*
Up stream	1.24	AA953_RS10405	*ybiC*
Up stream	1.39	AA953_RS10420	*ybiX*
Up stream	1.20	AA953_RS10440	*ybiO*
Up stream	1.40	AA953_RS10465	*dps*
Up stream	1.21	AA953_RS10485	*mntS*
Up stream	1.24	AA953_RS10555	*gsiB*
Up stream	1.32	AA953_RS10585	*bssR*
Up stream	1.33	AA953_RS10590	*yliI*
Up stream	1.09	AA953_RS10610	*ybjG*
Up stream	1.94	AA953_RS10665	*rimK*
Up stream	1.31	AA953_RS10715	*artM*
Up stream	1.17	AA953_RS10730	*artP*
Up stream	1.33	AA953_RS10850	*aat*
Up stream	1.21	AA953_RS10880	*lolA*
Up stream	1.11	AA953_RS10885	*rarA*
Up stream	1.13	AA953_RS10890	*serS*
Up stream	3.01	AA953_RS10900	*dmsB*
Up stream	1.21	AA953_RS10935	*pflA*
Up stream	1.31	AA953_RS10960	*serC*
Up stream	1.19	AA953_RS10985	*ihfB*
Up stream	1.18	AA953_RS11010	*ycaR*
Up stream	1.44	AA953_RS11020	*ycbJ*
Up stream	1.43	AA953_RS11050	*ldtD*
Up stream	1.34	AA953_RS11060	*ycbL*
Up stream	1.45	AA953_RS11065	*aspC*
Up stream	1.20	AA953_RS11180	*pqiA*
Up stream	1.19	AA953_RS11190	*pqiC*
Up stream	1.16	AA953_RS11215	*ompA*
Up stream	1.18	AA953_RS11225	*sulA*
Up stream	1.22	AA953_RS11285	*tusE*
Up stream	1.32	AA953_RS11365	*gfcD*
Up stream	1.29	AA953_RS11400	*cspG*
Up stream	1.22	AA953_RS11415	*yccM*
Up stream	1.23	AA953_RS11445	*torD*
Up stream	15.85	AA953_RS11550	*efeU*

Up stream	1.29	AA953_RS11570	*phoH*
Up stream	1.22	AA953_RS11570	*phoH*
Up stream	5.90	AA953_RS11585	*pgaB*
Up stream	1.38	AA953_RS11645	*ycdZ*
Up stream	1.16	AA953_RS11725	*opgH*
Up stream	1.26	AA953_RS11730	*yceK*
Up stream	2.66	AA953_RS11765	*yceJ*
Up stream	1.56	AA953_RS11800	*grxB*
Up stream	1.63	AA953_RS11895	*flgL*
Up stream	1.24	AA953_RS11935	*fabH*
Up stream	1.28	AA953_RS11940	*fabD*
Up stream	1.30	AA953_RS11950	*acpP*
Up stream	1.21	AA953_RS11960	*pabC*
Up stream	1.50	AA953_RS11985	*ptsG*
Up stream	60.24	AA953_RS12000	*ycfL*
Up stream	1.22	AA953_RS12015	*nagZ*
Up stream	1.20	AA953_RS12065	*lolD*
Up stream	1.18	AA953_RS12075	*nagK*
Up stream	1.19	AA953_RS12315	*ymgC*
Up stream	2.85	AA953_RS12320	*ycgG*
Up stream	3.44	AA953_RS12350	*ymgI*
Up stream	1.20	AA953_RS12360	*minE*
Up stream	1.20	AA953_RS12420	*dsbB*
Up stream	2.68	AA953_RS12470	*ymgE*
Up stream	1.25	AA953_RS12490	*dhaL*
Up stream	1.27	AA953_RS12500	*dhaR*
Up stream	1.52	AA953_RS12505	*ycgV*
Up stream	1.18	AA953_RS12535	*prs*
Up stream	1.38	AA953_RS12550	*hemA*
Up stream	1.27	AA953_RS12620	*narL*
Up stream	1.19	AA953_RS12695	*galU*
Up stream	1.17	AA953_RS12705	*tdk*
Up stream	1.12	AA953_RS12750	*oppF*
Up stream	1.27	AA953_RS12755	*yciU*
Up stream	1.20	AA953_RS12760	*cls*
Up stream	2.80	AA953_RS12780	*tonB*
Up stream	1.19	AA953_RS12795	*yciC*
Up stream	1.33	AA953_RS12955	*osmB*
Up stream	2.38	AA953_RS12965	*yciZ*
Up stream	1.11	AA953_RS13000	*sapF*
Up stream	1.32	AA953_RS13135	*ycjT*
Up stream	1.50	AA953_RS13150	*ompG*
Up stream	1.21	AA953_RS13160	*ycjX*
Up stream	1.74	AA953_RS13275	*smrA*

Up stream	1.22	AA953_RS13370	*ydaU*
Up stream	1.38	AA953_RS13395	*ynaK*
Up stream	1.52	AA953_RS13415	*lomR_1*
Up stream	2.72	AA953_RS13480	*hslJ*
Up stream	1.30	AA953_RS13550	*paaF*
Up stream	1.97	AA953_RS13565	*paaI*
Up stream	1.21	AA953_RS13575	*paaK*
Up stream	1.14	AA953_RS13620	*ydbD*
Up stream	1.42	AA953_RS13655	*ydcF*
Up stream	1.56	AA953_RS13660	*aldA*
Up stream	1.17	AA953_RS13680	*cybB*
Up stream	1.18	AA953_RS13690	*hokB*
Up stream	1.23	AA953_RS13705	*ydcJ*
Up stream	2.49	AA953_RS13760	*insQ*
Up stream	1.22	AA953_RS13815	*ydcV*
Up stream	3.14	AA953_RS13830	*ydcX*
Up stream	1.29	AA953_RS13855	*mcbR*
Up stream	24.43	AA953_RS13860	*yncD*
Up stream	1.26	AA953_RS13915	*nhoA*
Up stream	1.59	AA953_RS13945	*narU*
Up stream	1.15	AA953_RS13975	*fdnH*
Up stream	1.97	AA953_RS13990	*adhP*
Up stream	1.13	AA953_RS14055	*yddW*
Up stream	1.28	AA953_RS14080	*yddB*
Up stream	22.39	AA953_RS14085	*yddA*
Up stream	1.34	AA953_RS14090	*ydeM*
Up stream	1.15	AA953_RS14095	*ydeN*
Up stream	1.22	AA953_RS14105	*safA*
Up stream	1.15	AA953_RS14170	*lsrA*
Up stream	1.12	AA953_RS14175	*lsrC*
Up stream	1.24	AA953_RS14205	*yneE*
Up stream	1.42	AA953_RS14425	*AA953_RS14425*
Up stream	2.05	AA953_RS14550	*ynfC*
Up stream	1.28	AA953_RS14675	*rstB*
Up stream	1.15	AA953_RS14680	*tus*
Up stream	3.05	AA953_RS14800	*nth*
Up stream	1.67	AA953_RS15015	*ydhY*
Up stream	2.83	AA953_RS15065	*sufA*
Up stream	20.90	AA953_RS15180	*ydiE*
Up stream	3.05	AA953_RS15190	*ydiV*
Up stream	1.12	AA953_RS15235	*rplT*
Up stream	1.47	AA953_RS15370	*ves*
Up stream	2.75	AA953_RS15700	*rnd*
Up stream	5.59	AA953_RS15785	*yobD*

Up stream	1.29	AA953_RS15845	*htpX*
Up stream	3.63	AA953_RS15865	*yebS*
Up stream	1.46	AA953_RS15930	*ptrB*
Up stream	3.41	AA953_RS16225	*ftnB*
Up stream	2.91	AA953_RS16245	*ftnA*
Up stream	5.43	AA953_RS16305	*sdiA*
Up stream	1.55	AA953_RS16725	*yeeR*
Up stream	1.81	AA953_RS17135	*yegU*
Up stream	1.16	AA953_RS17400	*mglB*
Up stream	51.79	AA953_RS17435	*cirA*
Up stream	1.19	AA953_RS17440	*lysP*
Up stream	1.21	AA953_RS17720	*mqo*
Up stream	19.31	AA953_RS17725	*yojI*
Up stream	1.60	AA953_RS17765	*rcsB*
Up stream	2.22	AA953_RS17970	*arnD*
Up stream	4.44	AA953_RS18390	*yfcV*
Up stream	22.49	AA953_RS18670	*mntH*
Up stream	2.53	AA953_RS18970	*yffS*
Up stream	2.24	AA953_RS19070	*acrD*
Up stream	1.42	AA953_RS19080	*yffB*
Up stream	1.41	AA953_RS19320	*rodZ*
Up stream	1.29	AA953_RS19350	*sseB*
Up stream	1.20	AA953_RS19370	*hscA*
Up stream	1.36	AA953_RS19695	*pssA*
Up stream	1.19	AA953_RS19715	*rrfG*
Up stream	1.10	AA953_RS19725	*gltW*
Up stream	2.52	AA953_RS19850	*recN*
Up stream	1.60	AA953_RS20090	*ygaP*
Up stream	17.41	AA953_RS20120	*nrdI*
Up stream	1.22	AA953_RS20150	*ygaY*
Up stream	1.17	AA953_RS20160	*ygaH*
Up stream	1.48	AA953_RS20305	*norW*
Up stream	1.36	AA953_RS20360	*hycD*
Up stream	1.45	AA953_RS20460	*rpoS_C*
Up stream	1.87	AA953_RS20720	*yqcC*
Up stream	1.19	AA953_RS20755	*ygdG*
Up stream	1.22	AA953_RS20825	*tcdA*
Up stream	9.21	AA953_RS21165	*ygfK*
Up stream	1.31	AA953_RS21180	*xdhD*
Up stream	1.32	AA953_RS21185	*xanQ*
Up stream	1.24	AA953_RS21560	*yggN*
Up stream	1.22	AA953_RS21625	*yghF*
Up stream	1.34	AA953_RS21700	*AA953_RS21700*
Up stream	6.70	AA953_RS21805	*hybO*

Up stream	12.26	AA953_RS21845	*exbB*
Up stream	6.74	AA953_RS21855	*metC*
Up stream	1.34	AA953_RS21910	*parC*
Up stream	43.15	AA953_RS22185	*yqjH*
Up stream	40.20	AA953_RS22190	*yqjI*
Up stream	1.56	AA953_RS22365	*yhaJ*
Up stream	1.57	AA953_RS22395	*tdcF*
Up stream	1.32	AA953_RS22405	*tdcD*
Up stream	1.52	AA953_RS22455	*garP*
Up stream	1.23	AA953_RS22485	*agaV*
Up stream	1.19	AA953_RS22570	*yraP*
Up stream	1.21	AA953_RS22640	*nlpI*
Up stream	1.38	AA953_RS22685	*argG*
Up stream	2.79	AA953_RS22860	*yrbL*
Up stream	1.29	AA953_RS22890	*gltD*
Up stream	1.59	AA953_RS22950	*nanR*
Up stream	1.19	AA953_RS22990	*degQ*
Up stream	1.14	AA953_RS23005	*argR*
Up stream	1.19	AA953_RS23205	*rrfD*
Up stream	1.35	AA953_RS24615	*AA953_RS24615*
Up stream	1.27	AA953_RS24750	*AA953_RS24750*
Up stream	2.31	AA953_RS24895	*AA953_RS24895*
Up stream	31.60	AA953_RS25140	*AA953_RS25140*
Up stream	1.30	AA953_RS26285	*AA953_RS26285*
Up stream	1.30	AA953_RS26350	*AA953_RS26350*
Up stream	27.47	AA953_RS26415	*AA953_RS26415*
Up stream	8.01	AA953_RS26475	*AA953_RS26475*
Up stream	1.68	AA953_RS26600	*AA953_RS26600*
Down stream	1.09	AA953_RS00010	*AA953_RS00010*
Down stream	1.10	AA953_RS00025	*AA953_RS00025*
Down stream	1.11	AA953_RS00435	*yhfK*
Down stream	1.24	AA953_RS00440	*argD*
Down stream	1.23	AA953_RS00440	*argD*
Down stream	1.29	AA953_RS00555	*AA953_RS00555*
Down stream	1.60	AA953_RS00750	*rtcR*
Down stream	1.28	AA953_RS00860	*AA953_RS00860*
Down stream	1.36	AA953_RS00910	*livF*
Down stream	1.44	AA953_RS01290	*yhjE*
Down stream	1.21	AA953_RS01345	*bcsA*
Down stream	1.40	AA953_RS01385	*yhjV*
Down stream	1.15	AA953_RS01470	*yiaF*
Down stream	1.28	AA953_RS01555	*xylR*
Down stream	1.35	AA953_RS01560	*bax*
Down stream	1.25	AA953_RS01570	*avtA*

Down stream	1.25	AA953_RS01580	*ysaA*
Down stream	2.58	AA953_RS01675	*selA*
Down stream	1.22	AA953_RS01835	*rfaC*
Down stream	1.44	AA953_RS01905	*mutM*
Down stream	1.27	AA953_RS02360	*yieK*
Down stream	1.44	AA953_RS02395	*pstB*
Down stream	1.23	AA953_RS02560	*AA953_RS02560*
Down stream	1.12	AA953_RS02570	*AA953_RS02570*
Down stream	1.18	AA953_RS02585	*AA953_RS02585*
Down stream	1.18	AA953_RS02980	*ubiD*
Down stream	1.11	AA953_RS02985	*fre*
Down stream	1.19	AA953_RS02990	*fadA*
Down stream	1.23	AA953_RS03020	*AA953_RS03020*
Down stream	1.17	AA953_RS03035	*AA953_RS03035*
Down stream	1.09	AA953_RS03045	*mobB*
Down stream	1.48	AA953_RS03105	*yshB*
Down stream	1.10	AA953_RS03155	*yihO*
Down stream	1.22	AA953_RS03215	*fabY*
Down stream	1.40	AA953_RS03270	*frvR*
Down stream	1.25	AA953_RS03570	*eptC*
Down stream	1.29	AA953_RS03635	*murI*
Down stream	1.10	AA953_RS03650	*AA953_RS03650*
Down stream	1.32	AA953_RS03660	*murB*
Down stream	1.15	AA953_RS03750	*thiH*
Down stream	1.13	AA953_RS03820	*zraS*
Down stream	1.11	AA953_RS03860	*AA953_RS03860*
Down stream	1.27	AA953_RS03920	*pepE*
Down stream	1.14	AA953_RS04035	*ubiA*
Down stream	1.42	AA953_RS04085	*pspG*
Down stream	1.30	AA953_RS04105	*tyrB*
Down stream	1.52	AA953_RS04585	*yjeI*
Down stream	1.15	AA953_RS04815	*yjfN*
Down stream	1.44	AA953_RS04910	*ytfB*
Down stream	1.14	AA953_RS04925	*ytfE*
Down stream	1.28	AA953_RS04990	*tamB*
Down stream	1.22	AA953_RS05160	*AA953_RS05160*
Down stream	1.35	AA953_RS05215	*idnK*
Down stream	1.29	AA953_RS05230	*AA953_RS05230*
Down stream	1.94	AA953_RS05355	*yjhU*
Down stream	1.18	AA953_RS05540	*uxuB*
Down stream	1.14	AA953_RS05645	*yjiR*
Down stream	1.16	AA953_RS05660	*AA953_RS05660*
Down stream	1.31	AA953_RS05730	*lgoR*
Down stream	1.11	AA953_RS05850	*yjjW*

Down stream	1.24	AA953_RS05930	*trpR*
Down stream	1.19	AA953_RS06030	*mog*
Down stream	1.21	AA953_RS06080	*AA953_RS06080*
Down stream	1.10	AA953_RS06245	*pdxA*
Down stream	1.19	AA953_RS06325	*sgrR*
Down stream	1.33	AA953_RS06540	*ampE*
Down stream	1.13	AA953_RS06575	*acnB*
Down stream	1.14	AA953_RS06610	*hpt*
Down stream	1.17	AA953_RS06615	*can*
Down stream	1.23	AA953_RS06680	*htrE*
Down stream	1.72	AA953_RS06770	*erpA*
Down stream	1.32	AA953_RS06790	*dgt*
Down stream	1.13	AA953_RS07010	*AA953_RS07010*
Down stream	1.30	AA953_RS07035	*yafD*
Down stream	1.35	AA953_RS07045	*mltD*
Down stream	1.59	AA953_RS07095	*yafV*
Down stream	1.25	AA953_RS07320	*mmuM*
Down stream	1.12	AA953_RS07335	*AA953_RS07335*
Down stream	1.14	AA953_RS07410	*yagU*
Down stream	1.37	AA953_RS07520	*ykgG*
Down stream	1.15	AA953_RS07750	*yaiL*
Down stream	1.34	AA953_RS07790	*AA953_RS07790*
Down stream	1.59	AA953_RS07880	*yaiY*
Down stream	1.15	AA953_RS08030	*secD*
Down stream	1.19	AA953_RS08225	*ybaE*
Down stream	1.22	AA953_RS08380	*aes*
Down stream	1.13	AA953_RS08560	*glxK*
Down stream	1.22	AA953_RS08585	*ylbE*
Down stream	1.36	AA953_RS08640	*fimA*
Down stream	1.30	AA953_RS08890	*cusA*
Down stream	1.42	AA953_RS08895	*pheP*
Down stream	1.39	AA953_RS08900	*ybdG*
Down stream	1.36	AA953_RS08920	*ybdK*
Down stream	1.24	AA953_RS08960	*entF*
Down stream	1.23	AA953_RS09070	*uspG*
Down stream	1.28	AA953_RS09175	*ybeF*
Down stream	1.16	AA953_RS09225	*cobC*
Down stream	9.20	AA953_RS09255	*ybeQ*
Down stream	1.25	AA953_RS09340	*ubiF*
Down stream	1.24	AA953_RS09385	*nagD*
Down stream	1.36	AA953_RS09470	*AA953_RS09470*
Down stream	1.20	AA953_RS09590	*pxpC*
Down stream	2.68	AA953_RS09600	*nei*
Down stream	1.76	AA953_RS09680	*sucD*

Down stream	1.29	AA953_RS09740	*tolA*
Down stream	1.20	AA953_RS09800	*pnuC*
Down stream	1.23	AA953_RS09880	*ybhA*
Down stream	1.44	AA953_RS10395	*dinG*
Down stream	1.32	AA953_RS10405	*hcxB*
Down stream	1.14	AA953_RS10410	*ybiJ*
Down stream	1.18	AA953_RS10435	*rlmF*
Down stream	1.14	AA953_RS10440	*ybiO*
Down stream	2.10	AA953_RS10510	*ybiU*
Down stream	1.17	AA953_RS10565	*gsiD*
Down stream	1.10	AA953_RS10575	*dgcI*
Down stream	1.54	AA953_RS10580	*rimO*
Down stream	1.19	AA953_RS10605	*deoR*
Down stream	1.25	AA953_RS10670	*ybjN*
Down stream	1.27	AA953_RS10710	*artJ*
Down stream	1.23	AA953_RS10775	*hcp*
Down stream	1.23	AA953_RS10800	*AA953_RS10800*
Down stream	1.34	AA953_RS10875	*ftsK*
Down stream	1.19	AA953_RS10990	*ycaI*
Down stream	1.40	AA953_RS11020	*ycbJ*
Down stream	1.26	AA953_RS11045	*mukB*
Down stream	1.47	AA953_RS11090	*pepN*
Down stream	1.40	AA953_RS11170	*rlmKL*
Down stream	1.32	AA953_RS11205	*ycbZ*
Down stream	1.16	AA953_RS11230	*sxy*
Down stream	1.31	AA953_RS11235	*yccS*
Down stream	1.25	AA953_RS11390	*AA953_RS11390*
Down stream	1.12	AA953_RS11435	*torC*
Down stream	1.32	AA953_RS11560	*efeB*
Down stream	1.21	AA953_RS11575	*pgaD*
Down stream	1.25	AA953_RS11580	*pgaC*
Down stream	1.72	AA953_RS11595	*dgcT*
Down stream	1.12	AA953_RS11630	*ghrA*
Down stream	1.30	AA953_RS11705	*mdoC*
Down stream	1.13	AA953_RS11735	*msyB*
Down stream	1.17	AA953_RS11760	*yceI*
Down stream	1.19	AA953_RS11915	*yceF*
Down stream	1.19	AA953_RS11955	*fabF*
Down stream	1.14	AA953_RS12015	*nagZ*
Down stream	1.19	AA953_RS12080	*cobB*
Down stream	1.13	AA953_RS12110	*potA*
Down stream	1.13	AA953_RS12125	*roxA*
Down stream	1.18	AA953_RS12180	*lit*
Down stream	1.24	AA953_RS12290	*bluR*

Down stream	1.22	AA953_RS12360	*minE*
Down stream	1.23	AA953_RS12405	*hlyE*
Down stream	1.25	AA953_RS12450	*cvrA*
Down stream	1.23	AA953_RS12500	*dhaR*
Down stream	1.17	AA953_RS12525	*ychH*
Down stream	1.26	AA953_RS12530	*dauA*
Down stream	1.51	AA953_RS12615	*ychO*
Down stream	1.30	AA953_RS12620	*narL*
Down stream	1.25	AA953_RS12720	*adhE*
Down stream	1.15	AA953_RS12750	*oppF*
Down stream	1.50	AA953_RS12830	*trpCF*
Down stream	1.32	AA953_RS12900	*cysB*
Down stream	1.22	AA953_RS12940	*lapB*
Down stream	1.12	AA953_RS12945	*pyrF*
Down stream	1.31	AA953_RS13060	*puuB*
Down stream	1.31	AA953_RS13595	*AA953_RS13595*
Down stream	1.49	AA953_RS13695	*trg*
Down stream	1.21	AA953_RS13760	*insQ*
Down stream	1.19	AA953_RS13870	*ansP*
Down stream	1.21	AA953_RS14075	*pqqL*
Down stream	1.19	AA953_RS14110	*ydeP*
Down stream	1.61	AA953_RS14140	*hipA*
Down stream	1.18	AA953_RS14150	*AA953_RS14150*
Down stream	1.17	AA953_RS14160	*lsrK*
Down stream	1.13	AA953_RS14165	*lsrR*
Down stream	1.13	AA953_RS14170	*lsrA*
Down stream	1.45	AA953_RS14215	*uxaB*
Down stream	1.60	AA953_RS14280	*ydeE*
Down stream	1.38	AA953_RS14505	*AA953_RS14505*
Down stream	1.33	AA953_RS14545	*speG*
Down stream	2.98	AA953_RS14635	*tqsA*
Down stream	1.71	AA953_RS14680	*tus*
Down stream	1.67	AA953_RS14685	*fumC*
Down stream	1.58	AA953_RS15640	*dgcP*
Down stream	1.78	AA953_RS15755	*pdeD*
Down stream	1.67	AA953_RS15995	*mepM*
Down stream	1.78	AA953_RS16340	*fliC*
Down stream	1.39	AA953_RS16700	*AA953_RS16700*
Down stream	1.13	AA953_RS16865	*wzzB*
Down stream	1.24	AA953_RS16920	*AA953_RS16920*
Down stream	1.23	AA953_RS17400	*mglB*
Down stream	1.40	AA953_RS17575	*bcr*
Down stream	2.73	AA953_RS18305	*flk*
Down stream	1.52	AA953_RS18360	*yfcO*

Down stream	1.26	AA953_RS18545	*AA953_RS18545*
Down stream	1.85	AA953_RS19250	*pdeF*
Down stream	1.28	AA953_RS19340	*yfhM*
Down stream	1.27	AA953_RS19700	*yfiM*
Down stream	1.12	AA953_RS19720	*AA953_RS19720*
Down stream	1.90	AA953_RS19905	*abpA*
Down stream	1.35	AA953_RS19970	*AA953_RS19970*
Down stream	1.63	AA953_RS20650	*ygcG*
Down stream	1.17	AA953_RS20820	*csdE*
Down stream	1.60	AA953_RS20870	*ptrA*
Down stream	1.34	AA953_RS21075	*AA953_RS21075*
Down stream	1.24	AA953_RS21150	*yqeB*
Down stream	1.24	AA953_RS21515	*ruvX*
Down stream	1.12	AA953_RS21680	*glcC*
Down stream	1.45	AA953_RS21820	*AA953_RS21820*
Down stream	1.42	AA953_RS21865	*yqhC*
Down stream	1.38	AA953_RS22045	*AA953_RS22045*
Down stream	1.36	AA953_RS22055	*yqiH*
Down stream	1.30	AA953_RS22280	*alx*
Down stream	1.39	AA953_RS23040	*tldD*
Down stream	1.10	AA953_RS23210	*AA953_RS23210*
Down stream	1.17	AA953_RS23225	*AA953_RS23225*
Down stream	1.50	AA953_RS24865	*stfR*
Down stream	1.40	AA953_RS25655	*AA953_RS25655*
Down stream	1.34	AA953_RS25930	*AA953_RS25930*
Down stream	1.11	AA953_RS26285	*AA953_RS26285*
Down stream	1.21	AA953_RS26360	*yaiZ*

[a] "Up stream" means this peak covers the up stream of the CDS of a nearest gene and "Down stream" means that this peak covers the down stream of the CDS of a nearest gene.

Table S5.Transcriptional units identified to be controlled by Fur directly in this study.

Peak Fold enrichment[a]	Peak identification condition[b]	First gene of the transcriptional unit[c]	log_2 FC Fe+[d]	log_2 FC Fe-[e]	Mode of regulation[f]	Reference[g]
2.51	Fe+;Fe-	*bfr*	-2.02	-1.08	Activate	This work
2.51	Fe+;Fe-	*bfd*	1.49	non-DEG	Repress	Reported
1.38	Fe+	*tisB*	2.59	2.23	Repress	This work
1.27	Fe-	*rmuC*	0.64	non-DEG	Repress	Reported
1.95	Fe+;Fe-	*glnL*	non-DEG	-0.66	Activate	This work
6.00	Fe+;Fe-	*fecA*	2.34	1.16	Repress	Reported
12.35	Fe+;Fe-	*fecI*	3.32	non-DEG	Repress	Reported
1.50	Fe+;Fe-	*bglJ*	1.73	0.82	Repress	This work
56.70	Fe+;Fe-	*fhuF*	1.84	non-DEG	Repress	Reported
56.70	Fe+;Fe-	*yjjZ*	7.22	4.26	Repress	Reported
1.16	Fe-	*dapB*	non-DEG	1.08	Repress	This work
1.21	Fe+;Fe-	*leuD*	0.64	non-DEG	Repress	This work
33.06	Fe+;Fe-	*fhuA*	1.06	non-DEG	Repress	Reported
1.64	Fe+;Fe-	*fhuC*	1.19	non-DEG	Repress	Reported
1.34	Fe+	*dinB*	1.53	non-DEG	Repress	This work
1.33	Fe+;Fe-	*tsx*	-0.98	-0.69	Activate	Reported
6.99	Fe+;Fe-	*ybaN*	1.16	non-DEG	Repress	Reported
1.16	Fe+;Fe-	*entD*	4.64	non-DEG	Repress	Reported
46.07	Fe+;Fe-	*fepA*	5.74	non-DEG	Repress	Reported
46.07	Fe+;Fe-	*fes*	4.56	non-DEG	Repress	Reported
46.80	Fe+;Fe-	*fepD*	1.65	non-DEG	Repress	Reported
46.80	Fe+;Fe-	*entS*	1.97	non-DEG	Repress	Reported
46.81	Fe+;Fe-	*fepB*	2.41	non-DEG	Repress	Reported
46.80	Fe+;Fe-	*entC*	4.91	1.46	Repress	Reported
1.18	Fe+	*entB*	4.66	non-DEG	Repress	Reported
1.22	Fe+;Fe-	*entH*	4.22	non-DEG	Repress	Reported
1.19	Fe+	*sdhA*	-0.39	non-DEG	Activate	Reported
2.20	Fe+;Fe-	*sdhB*	-0.71	non-DEG	Activate	Reported
1.20	Fe-	*pnuC*	0.38	non-DEG	Repress	This work
14.73	Fe+;Fe-	*gpmA*	2.20	2.02	Repress	Reported
1.13	Fe+	*AA953_RS09935*	2.47	non-DEG	Repress	This work
1.12	Fe-	*TraR*	3.25	non-DEG	Repress	This work
1.35	Fe+;Fe-	*AA953_RS10025*	0.48	non-DEG	Repress	This work
1.35	Fe+;Fe-	*AA953_RS10030*	3.21	non-DEG	Repress	This work
1.24	Fe-	*AA953_RS10145*	3.57	non-DEG	Repress	This work
1.14	Fe+;Fe-	*bioC*	0.73	non-DEG	Repress	This work
1.21	Fe+;Fe-	*AA953_RS10300*	2.15	1.72	Repress	This work
1.21	Fe+;Fe-	*moaA*	1.95	1.82	Repress	This work

1.39	Fe+;Fe-	*ybiX*	4.68	non-DEG	Repress	Reported
27.47	Fe+;Fe-	*fiu*	5.14	non-DEG	Repress	Reported
27.47	Fe+;Fe-	*mcbA*	0.70	non-DEG	Repress	This work
1.21	Fe+;Fe-	*mntS*	-2.68	non-DEG	Activate	This work
1.94	Fe+;Fe-	*rimK*	0.51	non-DEG	Repress	This work
1.13	Fe-	*serS*	-0.30	non-DEG	Activate	This work
3.01	Fe+;Fe-	*dmsB*	-1.97	non-DEG	Activate	This work
1.16	Fe-	*AA953_RS11220*	1.15	non-DEG	Repress	This work
1.18	Fe+	*sulA*	2.82	non-DEG	Repress	This work
15.85	Fe+;Fe-	*efeO*	1.41	non-DEG	Repress	Reported
1.29	Fe-	*phoH*	0.62	non-DEG	Repress	This work
1.56	Fe+;Fe-	*grxB*	0.45	non-DEG	Repress	This work
1.63	Fe+;Fe-	*flgL*	-1.76	-1.60	Activate	This work
60.24	Fe+;Fe-	*fhuE*	4.05	non-DEG	Repress	Reported
2.68	Fe+;Fe-	*ycgR*	-1.57	-1.47	Activate	This work
1.12	Fe-	*oppF*	-0.96	-1.12	Activate	Reported
2.80	Fe+;Fe-	*yciI*	0.83	non-DEG	Repress	Reported
2.80	Fe+;Fe-	*tonB*	1.35	non-DEG	Repress	Reported
1.42	Fe+	*ydcF*	0.53	non-DEG	Repress	This work
24.43	Fe+;Fe-	*yncD*	0.63	non-DEG	Repress	Reported
24.43	Fe+;Fe-	*yncE*	2.91	1.15	Repress	Reported
1.59	Fe+	*narU*	-0.88	non-DEG	Activate	Reported
1.97	Fe+;Fe-	*adhP*	0.43	non-DEG	Repress	Reported
1.28	Fe+;Fe-	*yddB*	1.45	non-DEG	Repress	Reported
22.39	Fe+;Fe-	*yddA*	1.47	non-DEG	Repress	Reported
1.15	Fe-	*ydeN*	non-DEG	1.15	Repress	This work
2.83	Fe+;Fe-	*sufA*	3.28	1.41	Repress	Reported
20.90	Fe+;Fe-	*ydiE*	2.47	1.21	Repress	Reported
3.41	Fe+;Fe-	*araF*	-0.99	non-DEG	Activate	Reported
3.41	Fe+;Fe-	*ftnB*	-0.87	non-DEG	Activate	Reported
2.91	Fe+;Fe-	*ftnA*	-0.68	non-DEG	Activate	Reported
5.43	Fe+;Fe-	*sdiA*	-0.72	non-DEG	Activate	Reported
1.16	Fe-	*mglB*	-0.54	non-DEG	Activate	This work
51.79	Fe+;Fe-	*cirA*	6.06	non-DEG	Repress	Reported
1.19	Fe+	*lysP*	non-DEG	0.64	Repress	This work
1.21	Fe+	*mqo*	1.74	1.22	Repress	This work
19.31	Fe+;Fe-	*yojI*	0.58	non-DEG	Repress	Reported
22.49	Fe+;Fe-	*mntH*	1.33	2.39	Repress	Reported
1.29	Fe+	*sseB*	non-DEG	-0.70	Activate	This work
1.20	Fe+	*hscA*	-0.39	-0.59	Activate	This work
2.52	Fe+;Fe-	*recN*	3.19	non-DEG	Repress	Reported
17.40	Fe+;Fe-	*nrdH*	2.60	non-DEG	Repress	Reported
1.22	Fe-	*yghF*	-2.55	-2.19	Activate	This work
6.70	Fe+;Fe-	*hybO*	-1.44	non-DEG	Activate	Reported

6.70	Fe+;Fe-	*yghW*	-2.26	-4.64	Activate	This work
12.26	Fe+	*exbB*	1.90	0.85	Repress	Reported
43.15	Fe+	*yqjH (nfeF)*	2.27	1.29	Repress	Reported
40.20	Fe-	*yqjI (nfeR)*	1.05	non-DEG	Repress	This work
2.79	Fe+;Fe-	*yrbL*	0.62	non-DEG	Repress	Reported
1.13	Fe-	*mdh*	0.62	non-DEG	Repress	This work
1.14	Fe-	*argR*	0.78	non-DEG	Repress	This work

[a]The fold enrichment of Fur binding peaks were analyzed by MACS2 software. Each peak covered within -500 bp relative to the strat codon of the adjenct gene.

[b]Fur binding peak was identified in the growth condition with (Fe+) or without (Fe-) iron supplied.

[c]Symbol of the first gene in each of the transcriptional unit was shown.

[d]Fold changes of the first gene in each of the transcriptional unit in the transcriptome comparison of CY405 versus NCM3722 in the growht condition with iron supplied. The value was shown in Log_2. ‘non-DEG’ means that this gene is not differentially expressed.

[e]Same as that in c, but the data was obtained in the growth condition wihout iron supplied.

[f]‘Repress’ neans this gene was repssed by Fur and ‘Activate’ means this genes was activated by Fur.

[g]‘This work’ means this gene was newly identified in this work.‘Reported’ means this gene was a reported Fur regulon gene.

Table S6. Predicated binding sites of Fur on genes repressed by Fur.

Gene Symbol	Central relative position to start codon	Central relative position to TSS	Predicated binding sequence of Fur[a]	*p*-value
gpmA	-35	2	TAATGAGAATTATTATCATTA	3.38E-10
fhuF	-93	-4	ATATGATAATTTTTATCATTT	6.23E-10
yjjZ	-48	-21	AAATGATAAAAATTATCATAT	6.23E-10
mntH	-56	-28	ATTTGATAATCATTCTCGTTT	1.08E-09
fepA	-172	1	TATTGATAACTATTTGCATTT	1.99E-09
fes	-71	-9	AAATGCAAATAGTTATCAATA	1.99E-09
fhuA	-45	-3	TTATAATAATCATTCTCGTTT	2.81E-09
entC	-36	18	AAATGATAATCATTATTAAAG	6.30E-09
yqjH(*nfeF*)	-59	-12	GATTAACAATCATTATCATTT	9.08E-09
fecA	-79	-29	TAAGGAAAATAATTCTTATTT	2.99E-08
cirA	-189	-16	GATTGATAATTGTTATCGTTT	3.26E-08
exbB	-70	-40	TAATGAGAACGACTATCAATT	3.36E-08
sufA	-53	-21	AACTGATAATCATTATCACTA	3.54E-08
yncE	-91	-11	TAATGATTACCATTCCCATTT	3.57E-08
fepD	-25	-22	TAACGATAATTAATTTCATTA	4.10E-08
entS	-86	-39	TAATGAAATTAATTATCGTTA	4.10E-08
fiu	-123	-6	AAGTGATAATGCTTATCAAAA	5.44E-08
tonB	-54	-19	ATATGATTGCTATTTGCATTT	5.98E-08
fecI	-51	-4	TAATGATAACCATTCTCATAT	5.40E-07
yojI	-65	-39	AAATAAGAATTATTATTGCTG	1.19E-06
fhuE	-106	-10	GAATGCGTATATTTCTCATTT	1.37E-06
fepB	-218	-1	AAATGAGAAGCATTATTGATG	7.11E-06
tisB	-250	-34	AAATATTACTGTTTATTTATA	0.000159
dapB	-82	4	AATTTCTAATTATCAGCGTTT	0.000209
phoH	-93	-39	ATATAAGTAAATATATTGTTG	0.000439
moaA	-238	-21	GTAAAACCGCCATTTTCCCTT	0.000466
lysP	-15	13	TTTTTAAGATGGATAGCATTT	0.000734
yqjI(*nfeR*)	-70	-44	TAAAAATCATTTTTACACTTG	0.000831
mdh	-257	-55	AAAGTAAATTAATTGTTATCA	9.19E-05
recN	-93	-57	TCAAAAAAATTATTCTAATTT	0.000295
argR	-178	-77	TGATAACAATTAATTTACTTT	9.19E-05
mcbA	-210	-113	ATCTGGCTGCGTTTATCATTC	0.000381
yrbL	-145	-114	GTACTGAAATCGTTCTCAATC	0.000363
yciI	-170	-143	AAATGCAAATAGCAATCATAT	5.98E-08
rimK	-246	-155	GCATGAAAACAGCTATCAACC	0.000597
sulA	-185	-157	ATAAAAGAATGATTCACATTA	1.12E-06
nrdH	-238	-171	GTGCAAAAATGATAATAAATA	0.000437
ydeN	-204	-174	AAATGCAATAATTTATTAAAT	5.64E-07
bfd	-40	nd	AAATAAGAACTATTTTCATTT	7.61E-10

yncD	-151	nd	AAATGGGAATGGTAATCATTA	3.57E-08
ybaN	-49	nd	CAATGAAAATGATAATTGTTA	1.51E-07
ydiE	-29	nd	TAATAAGAATCATTGTTATAT	1.69E-07
mqo	-119	nd	AAATTATAATGTTTCTAAAAT	1.20E-06
yddA	-287	nd	TAATTAATAACGCTCTCTTTT	7.55E-05
fepE	-137	nd	GTTTGATGAATATTTCTCTTA	0.000181
AA953_RS10030	-275	nd	TTATAAGCATTTAATGCATTG	0.000187
yddB	-243	nd	GAAAAACAACGTATCGCCCTG	0.000351
ydcF	-134	nd	TAATGTTTATCTACAGCATTT	0.000358
ybiX	-104	nd	CAACAAGAGCGGCTACCGTTA	0.000466
AA953_RS11220	-211	nd	CGATAGCTGTCTTTTTCATTT	0.000752
AA953_RS10025	-229	nd	CAGCGTTTATAGTTAAAAAAA	0.000862
AA953_RS09935	-271	nd	CCTTTTTTATTATTCGCATTC	0.000974
TraR	-53	nd	CCTTTTTTATTATTCGCATTC	0.000974

[a]Binding site was predicated by MEME and for those with multiple Fur binding sites predicted, the binding sequence with the lowest p-value is shown.

Table S7. Predicated binding sites of Fur on genes activated by Fur.

Gene Symbol	Central relative position to start codon	Central relative position to TSS	Predicated binding sequence of Fur[a]	*p*-value
araF	-318	-208	CTGGTTAAAAAGAAG	2.69E-07
hybO	-274	-172	CTGGATAAAGAGATG	3.07E-06
yghF	-319	-169	CTGGCGAAAAAACAG	2.91E-05
mglB	-365	-147	CACTGTTTTATGCCG	9.97E-06
ftnA	-136	-102	CTGATTAAAGAAATA	1.40E-05
tsx	-309	-77	CAACCTGTTATGCAG	5.13E-06
sdiA	-158	-73	CTGCTTAACAAATCA	1.60E-05
ycgR	-97	-68	CTGATTTAAGAACAA	0.0002
serS	-127	-64	TGGCTGAACAGGATC	0.00173
ftnB	-208	-58	CATTGATTTCATCAA	5.97E-05
narU	-108	-44	TATTCTTTCAATCAG	2.39E-05
flgL	-91	nd	CAACGTTTTCAGCAG	5.28E-08
sdhB	-221	nd	CTGATGGAAACGGCG	1.01E-06
bfr	-338	nd	TTGCTTAAAAAAGAG	3.71E-06
dmsB	-21	nd	CAGGTTGAAAAGGTG	5.18E-06
hscA	-252	nd	CTGATGGAACAGTTG	7.53E-06
oppF	-202	nd	TTGCTCAACGCGGTG	1.12E-05
sdhA	-182	nd	CTCTGCGTTCACCAA	2.52E-05
mntS	-129	nd	CACATTGTAAACCAG	2.60E-05
sseB	-316	nd	CGGCGTATCCGGCAG	0.000111

[a]Binding site was predicated by MEME and for those with multiple Fur binding sites predicted, binding sequence with the lowest *p*-value is shown.

Table S8. Fold changes of DEGs directly controlled by Fur identified in the transcriptome comparison of CY405 versus NCM3722 grown in the conditions with or without iron.

Gene ID	Gene Symbol	Log_2 FC (Fe-)	Log_2 FC (Fe+)	*q*-value (Fe-)	*q*-value (Fe+)
AA953_RS02120	*tisB*	2.23	2.59	0.0069	6.51E-15
AA953_RS05325	*fecA*	1.16	2.34	0.0065	3.87E-53
AA953_RS05775	*bglJ*	0.82	1.73	0.0008	5.35E-21
AA953_RS08050	*tsx*	-0.69	-0.98	0.0003	7.37E-14
AA953_RS08995	*entC*	1.46	4.91	0.0086	7.77E-35
AA953_RS09825	*gpmA*	2.02	2.20	1.0552E-22	2.72E-82
AA953_RS11895	*flgL*	-1.60	-1.76	3.4462E-14	3.80E-23
AA953_RS12465	*ycgR*	-1.47	-1.57	0.000033058	1.91E-09
AA953_RS12750	*oppF*	-1.12	-0.96	0.0011	2.34E-07
AA953_RS13865	*yncE*	1.15	2.91	0.0075	3.50E-150
AA953_RS15065	*sufA*	1.41	3.28	1.0607E-06	3.28E-123
AA953_RS15180	*ydiE*	1.21	2.47	0.0027	1.93E-36
AA953_RS17720	*mqo*	1.22	1.74	0.000044852	6.38E-11
AA953_RS18670	*mntH*	2.39	1.33	0.0043	4.91E-20
AA953_RS19370	*hscA*	-0.59	-0.39	0.0014	0.004
AA953_RS21625	*yghF*	-2.19	-2.55	0.0047	0.0003
AA953_RS21845	*exbB*	0.85	1.90	0.0097	9.83E-54
AA953_RS22185	*yqjH*	1.29	2.27	5.9996E-07	8.81E-71

Table S9. Fold changes of DEGs directly controlled by Fur identified in the transcriptome comparison of CY405 versus NCM3722 grown in the condition with iron.

GeneID	Gene symbol	Log_2 FC	ABS (Log_2 FC)	*q*-value	Fur regulation
AA953_RS00320	*bfd*	1.49	1.49	4.69E-08	Repress
AA953_RS02120	*tisB*	2.59	2.59	6.51E-15	Repress
AA953_RS02935	*rmuC*	0.64	0.64	7.37E-06	Repress
AA953_RS05325	*fecA*	2.34	2.34	3.87E-53	Repress
AA953_RS05335	*fecI*	3.32	3.32	2.26E-88	Repress
AA953_RS05775	*bglJ*	1.73	1.73	5.35E-21	Repress
AA953_RS05780	*fhuF*	1.84	1.84	5.86E-35	Repress
AA953_RS05785	*yjjZ*	7.22	7.22	0	Repress
AA953_RS06340	*leuD*	0.64	0.64	3.76E-07	Repress
AA953_RS06740	*fhuA*	1.06	1.06	5.24E-18	Repress
AA953_RS06745	*fhuC*	1.19	1.19	1.43E-12	Repress
AA953_RS07155	*dinB*	1.53	1.53	9.09E-12	Repress
AA953_RS08340	*ybaN*	1.16	1.16	1.01E-06	Repress
AA953_RS08940	*entD*	4.64	4.64	1.40E-105	Repress
AA953_RS08945	*fepA*	5.74	5.74	3.81E-34	Repress
AA953_RS08950	*fes*	4.56	4.56	4.44E-153	Repress
AA953_RS08980	*fepD*	1.65	1.65	7.73E-32	Repress
AA953_RS08985	*entS*	1.97	1.97	3.49E-43	Repress
AA953_RS08990	*fepB*	2.41	2.41	1.61E-120	Repress
AA953_RS08995	*entC*	4.91	4.91	7.77E-35	Repress
AA953_RS09005	*entB*	4.66	4.66	1.86E-150	Repress
AA953_RS09015	*entH*	4.22	4.22	1.70E-79	Repress
AA953_RS09800	*pnuC*	0.38	0.38	0.0083	Repress
AA953_RS09825	*gpmA*	2.20	2.20	2.72E-82	Repress
AA953_RS09935	AA953_RS09935	2.47	2.47	6.89E-33	Repress
AA953_RS09940	*TraR*	3.25	3.25	9.39E-08	Repress
AA953_RS10025	AA953_RS10025	0.48	0.48	0.0002	Repress
AA953_RS10030	AA953_RS10030	3.21	3.21	1.10E-68	Repress
AA953_RS10145	AA953_RS10145	3.57	3.57	1.77E-80	Repress
AA953_RS10280	*bioC*	0.73	0.73	1.12E-05	Repress
AA953_RS10300	AA953_RS10300	2.15	2.15	2.56E-11	Repress
AA953_RS10305	*moaA*	1.95	1.95	1.72E-78	Repress
AA953_RS10420	*ybiX*	4.68	4.68	7.04E-123	Repress
AA953_RS10425	*fiu*	5.14	5.14	1.58E-167	Repress
AA953_RS10430	*mcbA*	0.70	0.70	0.0023	Repress
AA953_RS10665	*rimK*	0.51	0.51	0.0092	Repress
AA953_RS11220	AA953_RS11220	1.15	1.15	2.41E-06	Repress
AA953_RS11225	*sulA*	2.82	2.82	3.35E-149	Repress
AA953_RS11555	*efeO*	1.41	1.41	4.38E-32	Repress

AA953_RS11570	*phoH*	0.62	0.62	0.0012	Repress
AA953_RS11800	*grxB*	0.45	0.45	0.0005	Repress
AA953_RS11990	*fhuE*	4.05	4.05	3.39E-193	Repress
AA953_RS12775	*yciI*	0.83	0.83	1.10E-05	Repress
AA953_RS12780	*tonB*	1.35	1.35	3.68E-31	Repress
AA953_RS13655	*ydcF*	0.53	0.53	0.0015	Repress
AA953_RS13860	*yncD*	0.63	0.63	0.0005	Repress
AA953_RS13865	*yncE*	2.91	2.91	3.50E-150	Repress
AA953_RS13990	*adhP*	0.43	0.43	9.84E-05	Repress
AA953_RS14080	*yddB*	1.45	1.45	1.92E-29	Repress
AA953_RS14085	*yddA*	1.47	1.47	6.66E-15	Repress
AA953_RS15065	*sufA*	3.28	3.28	3.28E-123	Repress
AA953_RS15180	*ydiE*	2.47	2.47	1.93E-36	Repress
AA953_RS17435	*cirA*	6.06	6.06	2.11E-274	Repress
AA953_RS17720	*mqo*	1.74	1.74	6.38E-11	Repress
AA953_RS17725	*yojI*	0.58	0.58	3.33E-05	Repress
AA953_RS18670	*mntH*	1.33	1.33	4.91E-20	Repress
AA953_RS19850	*recN*	3.19	3.19	4.17E-107	Repress
AA953_RS20115	*nrdH*	2.60	2.60	7.51E-53	Repress
AA953_RS21845	*exbB*	1.90	1.90	9.83E-54	Repress
AA953_RS22185	*yqjH*	2.27	2.27	8.81E-71	Repress
AA953_RS22190	*yqjI*	1.05	1.05	1.46E-09	Repress
AA953_RS22860	*yrbL*	0.62	0.62	2.93E-05	Repress
AA953_RS23000	*mdh*	0.62	0.62	2.71E-06	Repress
AA953_RS23005	*argR*	0.78	0.78	4.16E-12	Repress
AA953_RS00315	*bfr*	-2.02	2.02	3.697E-70	Activate
AA953_RS08050	*tsx*	-0.98	0.98	7.374E-14	Activate
AA953_RS09650	*sdhA*	-0.39	0.39	0.0012	Activate
AA953_RS09655	*sdhB*	-0.71	0.71	1.277E-08	Activate
AA953_RS10485	*mntS*	-2.68	2.68	0.0032	Activate
AA953_RS10890	*serS*	-0.30	0.30	0.0086	Activate
AA953_RS10900	*dmsB*	-1.97	1.97	0.0063	Activate
AA953_RS11895	*flgL*	-1.76	1.76	3.796E-23	Activate
AA953_RS12465	*ycgR*	-1.57	1.57	1.914E-09	Activate
AA953_RS12750	*oppF*	-0.96	0.96	2.34E-07	Activate
AA953_RS13945	*narU*	-0.88	0.88	0.0043	Activate
AA953_RS16220	*araF*	-0.99	0.99	7.288E-05	Activate
AA953_RS16225	*ftnB*	-0.87	0.87	3.351E-10	Activate
AA953_RS16245	*ftnA*	-0.68	0.68	0.0002	Activate
AA953_RS16305	*sdiA*	-0.72	0.72	1.481E-05	Activate
AA953_RS17400	*mglB*	-0.54	0.54	0.0043	Activate
AA953_RS19370	*hscA*	-0.39	0.39	0.004	Activate
AA953_RS21625	*yghF*	-2.55	2.55	0.0003	Activate
AA953_RS21805	*hybO*	-1.44	1.44	2.934E-05	Activate

AA953_RS21815	*yghW*	-2.26	2.26	0.0051	Activate

Table S10. Strains used in this work.

Strain	Description	Reference
**NCM3722 derivatives**		
NCM3722	wild type	Lab stock
CY405	△*fur*::kan, Kan^r	This work
CY598	FliC(K87N) *fliT*::kan, Kan^r	This work
CY856	FliC(K87N) *fliT*::kan, △*fur*::cm, Kan^rCm^r	This work
CY1099	*fur*-6×his-kan, Kan^r	This work
**MG1655 derivatives**		
MG1655	wild type	Lab stock
CY857	△*fur*::cm, Cm^r	This work
**BL21 derivatives**		
CY668	pET24a-*fur*, Kan^r	Lab stock

Table S11. Primers used in EMSA assay in this study.

Promoter	Primer	sequence	Size (bp)[a]
P*frdA*	PfrdA-F	GCCAGGATGCCGTTTCGCTCATAG	371
	PfrdA-R	CAAGATCGGCTTGAAAGGTTTGC	
P*nuoA*	PnuoA-F2	CTCTCTTTTGGGGGGAGGAATCG	379
	PnuoA-R2	CTTCAGTGGATGTTGACATACTC	
P*bfr*	Pbfr-F	CGTTTGTCTTTGTAATGGTATCAG	303
	Pbfr-R	CAGTTTGTTGAGATAATTTATAAC	
P*bfd*	Pbfd-F	GTTGGTATCTGGTCAATCGC	300
	Pbfd-R	AGAGACTCCCGTATACTTTC	
P*leuD*	QleuD-F	AACTCGCGCATTGAAGATTT	367
	QleuD-R	GGTGTGCTCCTTATTTAATGTTGCG	
P*moaA*	PmoaA-F	TTCTTCTACCTCTAAAGGAC	304
	PmoaA-R	GTACACCTTTCCAGATACGG	
P*pnuC*	QpnuC-F	CGAAAACATTGCCACATCAG	333
	QpnuC-R	CTTTCCCCCAAAGCGTAA	
P*dinB*	QdinB-F	ATGCGATGGCCTACAAAAAC	387
	QdinB-R	TGCTCACCTCTCAACACTGGTAA	
P*sulA*	QsulA-F	CAGGCTGTAACTGCCAAACA	371
	QsulA-R	AATCAATCCAGCCCCTGTGA	
P*serS*	QserS-F	GCCGACGAAATTAATGAAGG	310
	QserS-R	GCTTATCCTGTGCTTATCGAAT	
P*rimK*	QrimK-F	ATTGAAGCGGTGACGAAACT	378
	QrimK-R	CCTGCACCTCTCTGTCAGACC	
P*ydeN*	QydeN-F	CACACACGCTGCGTAAGAAT	378
	QydeN-R	AAATGTTCCTCTTCTTATTGTACTT	
P*yddA*	PyddA-F	CCATTCTTAGCCTCCTTCTTATG	302
	PyddA-R	CATACGAAGCGTAATGGGAATGG	

[a]length of the fragment.

Table S12. Primers of RT-qPCR used in this study.

Gene	Primer	Sequence
flhD	Q-flhD-F	TCCGCAAATGGTTAAGCTGG
	Q-flhD-R	AGCGTGTTGAGAGCATGATG
flhC	Q-flhC-F	CTGCCATTCTCAACCGACTG
	Q-flhC-R	TCGACGCCATTACACAAACC
fliC	Q-fliC-F	ATCTTTACGCTGCGGATGTG
	Q-fliC-R	CGACCACTTCTGTTTTGCCA
flgD	Q-flgD-F	CCAGTCGTTACAGGCCAGTA
	Q-flgD-R	GTAACTTTGTCTGCCGCCTG
pdeH	Q-pdeH-F	CTGGTGGAGCATATCCGTCT
	Q-pdeH-R	ACTGACGCAGCATCACAAAC
fliA	Q-fliA-F	TGAAGTGGCACAGGCAATAG
	Q-fliA-R	TGGCTGTTATTGGTGTCGAG
flgA	Q-flgA-F	GCTGGCAATGTCAAACTGAA
	Q-flgA-R	GCGAAACTGGGTTAACTGGA
